# Inhibition of DNA Topoisomerase Ι by Flavonoids and Polyacetylenes Isolated from *Bidens pilosa* L.

**DOI:** 10.3390/molecules29153547

**Published:** 2024-07-27

**Authors:** Guiyuan Zeng, Yinyue Wang, Meihua Zhu, Jumei Yi, Junjie Ma, Bijuan Yang, Weiqing Sun, Fang Dai, Junlin Yin, Guangzhi Zeng

**Affiliations:** 1 Key Laboratory of Chemistry in Ethnic Medicinal Resources, State Ethnic Affairs Commission and Ministry of Education, Yunnan Minzu University, Kunming 650504, China; yuan8556@126.com (G.Z.); wyy13995611885@163.com (Y.W.); zhm1773@163.com (M.Z.); 18468119534@163.com (J.Y.); mjj1336042031@163.com (J.M.); bijuan051322@163.com (B.Y.); weiqingsun1985@163.com (W.S.); 2 Yunnan Key Laboratory of Chiral Functional Substance Research and Application, Yunnan Minzu University, Kunming 650504, China; 3 School of Chemistry and Environmental Engineering, Qujing Normal University, Qujing 655011, China; daifangld@163.com

**Keywords:** *Bidens pilosa* L., flavonoids, polyacetylenes, cytotoxicity, DNA topoisomerase I, structure–activity analysis

## Abstract

Human DNA topoisomerase I (Topo I) is an essential enzyme in regulating DNA supercoiling during transcription and replication, and it is an important therapeutic target for anti-tumor agents. *Bidens pilosa* L. is a medicinal herb that is used as a folk medicine for cancers in China. A new flavonoid (**1**) and a new polyacetylene (**20**), along with eighteen flavonoids (**2**–**19**) and nine polyacetylenes (**21**–**29**), were isolated and identified from the methanol extract of the whole plant of *B. pilosa*, and some of the compounds (**4**, **5**, **6** and **7**) exhibited potent cytotoxicity against a panel of five human cancer cell lines. The DNA relaxation assay revealed that some flavonoids and polyacetylenes exerted inhibitory activities on human DNA Topo I, among them compounds **1**, **2**, **5**, **6**, **7**, **8**, **15**, **19**, **20**, **22**, and **24** were the most active ones, with IC_50_ values of 393.5, 328.98, 145.57, 239.27, 224.38, 189.84, 89.91, 47.5, 301.32, 178.03, and 218.27 μM, respectively. The structure–activity analysis of flavonoids was performed according to the results from the Topo I inhibition assay. The DNA content analysis revealed that **5**, **6**, and **7** potently arrested cell cycle at the G_1_/S and G_2_/M phases in human colon cancer cell DLD-1 depending on the concentration of the inhibitors. The levels of protein expression related to the G_1_/S and G_2_/M cell cycle checkpoints were in accordance with the results from the DNA content analysis. These findings suggest that flavonoids are one of the key active ingredients accounting for the anti-tumor effect of *B. pilosa*.

## 1. Introduction

*Bidens pilosa* L. is an annual herb of the Asteraceae; it is edible and has been traditionally used for the treatment of many diseases in many countries. For its anti-hyperglycemic, antihypertensive, and anti-tumor effects, *B. pilosa* has been widely used as a folk herbal medicine in China for treating various disorders, and in Brazil, it has been included in the official list of medicinal plants by the public health system [1]. Modern phytochemical studies revealed that *B. pilosa* contains flavonoids, polyacetylenes, phenolic acids, terpenoids, steroids, and other components [2], of which flavonoids and polyacetylenes are the main constituents [3]. A wide range of biological activities has been reported about *B. pilosa* and the compounds isolated from it, including antioxidative, anti-inflammatory, antibacterial, anticancer, antidiabetic, and immunomodulatory activities, etc. [4,5]. Flavonoids are a kind of polyphenols that broadly exist in plants and exhibit a wide range of bioactivities; to date, more than one hundred flavonoids have been isolated from *B. pilosa,* which were reported to have antioxidant, hepatoprotective, and cytotoxic activities, etc. [6]. Acetylenes are a kind of compounds containing one or more carbon–carbon triple bond(s) in their backbones and were found regularly in only five families, namely Campanulaceae, Asteraceae, Araliaceae, Pittosporaceae, and Umbelliferae. To date, more than fifty acetylenes have been isolated from *B. pilosa*, which are reported to play an important role in the plant with anti-cancer, anti-malaria, and anti-diabetes activities [7,8].

DNA topoisomerases (Topos) are the key enzymes that control DNA topology through the passage of DNA strands [9], which is important for DNA replication and transcription. According to whether they make transient single- or double-stranded breaks in the DNA, Topos are classified as Topo I and II, respectively [10]. Because of the overexpression and higher activity in tumor cells compared to normal cells, Topos have become important therapeutic targets for anti-tumor drugs [11]. Many natural compounds isolated from plants, such as camptothecin (CPT) and podophyllotoxin, exhibit potent inhibitory activities in Topos [12], and their derivatives are used as important anti-tumor drugs in the clinic, such as topotecan, irinotecan, and etoposide [13].

Currently, many studies have been conducted on the anti-tumor potential of natural products against Topos. As far as we know, no description of the inhibitory effect of polyacetylenes on Topos has been found, and the mechanism of the *B. pilosa* compounds’ activity has not been elucidated. Regarding the good anticancer activity of *B. pilosa*, the chemical and pharmacological constituents of the plant were investigated by us [14]. In this study, a new flavonoid and a new polyacetylene, together with 27 known flavonoids and polyacetylenes, were isolated from *B. pilosa*, of which some showed potent cytotoxicity and inhibitory activities on DNA Topo I. In this paper, we report the identification of the new compounds, the cytotoxicity, the inhibitory activities on Topo I, and the cytotoxic mechanism in cancer cells. To the best of our knowledge, this is the first time that the inhibition of flavonoids and polyacetylenes isolated from *B. pilosa* against DNA Topo I is reported.

## 2. Results and Discussion

### 2.1. Identification of Two New Compounds

**Figure 1:**
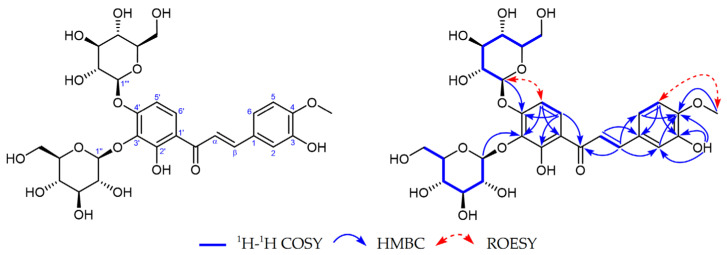
Key ^1^H–^1^H COSY, HMBC, and ROESY correlations of compound **1**.

Compound **1**, obtained as a yellow powder, had the molecular formula of C_28_H_34_O_16_ by the positive HR-ESI-MS (*m*/*z* 649.1746 [M + Na]^+^, calcd. 649.1739), requiring 12 degrees of unsaturation (Figure S1). The UV spectrum (Figure S9) exhibited a maximum absorption peak at 372 nm, which was typical of chalcones. The ^1^H NMR spectrum in DMSO-*d6* (Figure S2 and Table 1) showed one hydrogen-bonded phenolic hydroxy at *δ*H 12.90 (1H, br s, 2′-OH); one normal phenolic hydroxy at *δ*H 9.25 (1H, s, 3-OH); a pair of mutually coupled aromatic doublets at *δ*H 8.02 (1H, d, *J* = 9.3 Hz, H-6′) and 6.84 (1H, d, *J* = 9.3 Hz, H-5′) due to a 1,2,3,4-tetrasubstituted benzene ring; a set of 1,3,4-trisubstituted benzene ring signals at *δ*H 7.34 (1H, d, *J* = 1.9 Hz, H-2), 7.32 (1H, dd, *J* = 8.4, 1.9 Hz, H-6), and 7.01 (1H, d, *J* = 8.4 Hz, H-5); a pair of characteristic trans-olefinic doublets at *δ*H 7.70 and 7.77 (each 1H, d, *J* = 15.4 Hz); and two *β*-glucopyranosyl anomeric protons at *δ*H 4.98 (1H, d, *J* = 7.4 Hz, H-1‴) and 4.85 (1H, d, *J* = 7.6 Hz, H-1″), together with a series of other overlapped signals from the sugar moieties, as well as a methoxy signal at *δ*H 3.84 (3H, s). The ^13^C NMR spectrum (Figure S3 and Table 1) showed a total of 28 carbon resonances, including a conjugated ketone carbonyl carbon at *δ*C 192.1 (s), 14 aromatic or olefinic carbons due to two benzene rings and a double bond group, two sugar anomeric carbons at *δ*C 103.8 (d, C-1″) and 100.7 (d, C-1‴), combined with five pairs of characteristic oxygenated carbons (8 × CH, 2 × CH_2_) assignable to two *β*-glucopyranosyl moieties, as well as a methoxy carbon at *δ*C 55.8 (q). The existence of two benzene rings, one carbonyl, one double bond, and two sugar rings was exactly matched with its degrees of unsaturation. The above NMR features are generally similar to those of 4-*O*-methylokanin 4′-O-*β*-d-glucopyranoside, a chalcone glycoside isolated from a plant of the same genus [15]. The careful comparison of their NMR data revealed that Compound **1** had an extra *β*-glucopyranosyl moiety. A detailed analysis of the HMBC correlations (Figure 1 and Figure S6) confirmed that the aglycone structure was the same as that of 4-*O*-methylokanin 4′-*O*-*β*-d-glucopyranoside. It should be pointed out that the position of the methoxy group was also verified by the ROESY correlation (Supplemental Figure S8; Figure 1) of OCH_3_↔H-5. Furthermore, the HMBC correlations from H-6′ [*δ*H 8.02 (1H, d, *J* = 9.3 Hz)] and H-1‴ [*δ*H 4.98 (1H, d, *J* = 7.4 Hz)] to C-4′ [*δ*C 155.6 (s)] indicated the presence of a *β*-glucopyranosyl at C-4′, which was also supported by the ROESY correlation of H-1‴ ↔ H-5′. The HMBC correlations from H-5′ [*δ*H 6.84 (1H, d, *J* = 9.3 Hz)] and H-1″ [*δ*H 4.85 (1H, d, *J* = 7.6 Hz)] to C-3′ [*δ*C 133.6 (s)] positioned the other *β*-glucopyranosyl at C-3′. Therefore, the structure of Compound **1** was established as 4-*O*-methylokanin 3′,4′-di-*O*-*β*-d-glucopyranoside, shown in Figure 1.

**Figure 2:**
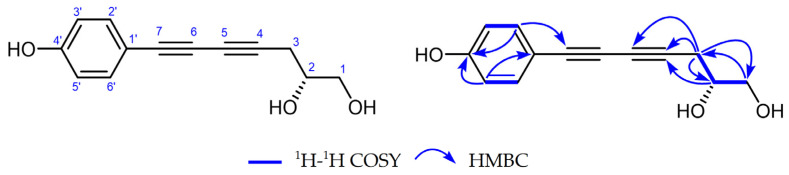
Key ^1^H–^1^H COSY and HMBC correlations of compound **20**.

Compound **20**, obtained as a white powder, had a molecular formula of C_13_H_12_O_3_ by the positive HR-ESI-MS (*m*/*z* 217.0865 [M + H]^+^, calcd. 217.0859), requiring eight degrees of unsaturation (Figure S11). In the IR spectrum, absorption bands attributable to the acetylene (2146 cm^−1^) and hydroxyl (3269, 3396 cm^−1^) groups were observed (Figure S19). The ^1^H NMR spectrum (Figure S12 and Table 2) in CD_3_OD showed a pair of mutually coupled aromatic doublets at *δ*H 7.28 (2H, d, *J* = 8.6 Hz, H-2′, H-6′) and 6.72 (2H, d, *J* = 8.6 Hz, H-3′, H-5′), corresponding to a p-substituted benzene ring, an oxygenated methine signal at *δ*H 3.77 (1H, dddd, *J* = 6.2, 6.0, 5.8, 4.9 Hz, H-2), an oxygenated methylene signal at *δ*H 3.54 (1H, dd, *J* = 11.2, 5.8 Hz, H-1a) and 3.59 (1H, dd, *J* = 11.2, 4.9 Hz, H-1b), as well as an aliphatic methylene signal at *δ*H 2.50 (1H, dd, *J* = 17.3, 6.2 Hz, H-3a) and 2.60 (1H, dd, *J* = 17.3, 6.0 Hz, H-3b), which means the existence of three exchangeable protons in the structure. The ^13^C NMR spectrum (Figure S13 and Table 2) showed a total of 11 carbon signals, including 4 aromatic carbons assignable to a p-substituted benzene ring, 4 characteristic quaternary carbons at *δ*C 80.8 (s, C-4), 76.2 (s, C-7), 73.2 (s, C-6) and 67.6 (s, C-5) due to two acetylenic groups, an oxygenated methine carbon at *δ*C 71.7 (d, C-2), an oxygenated methylene carbon at *δ*C 66.0 (t, C-1), as well as an aliphatic methylene carbon at *δ*C 25.2 (t, C-3). The analysis of the degrees of unsaturation also supported the presence of two acetylenic groups. The above NMR features were generally similar to those of 7-phenylhepta-4,6-diyne-1,2-diol, a polyacetylene isolated from the same plant [3]. The comparison of their NMR data revealed that the obvious difference only came from the substitution pattern of the benzene ring. The HMBC correlations (Figure S16 and Figure 2) from H-2′ and H-6′ [*δ*H 7.28 (2H, d, *J* = 8.6 Hz)] to C-4′ [*δ*C 160.0 (s)] indicated the presence of a phenolic hydroxy group at C-4′. The resulting structure was further verified by the 2D NMR analysis. The absolute configuration of C-2 was tentatively deduced to be R-form by the comparison of the specific rotatory values with synthetic analogues [16]. Finally, the structure of Compound **20** was established as (*R*)-7-(4-hydroxyphenyl) hepta-4,6-diyne-1,2-diol, shown in Figure 2.

### 2.2. Structures of Compounds ***1**–**29***

**Figure 3:**
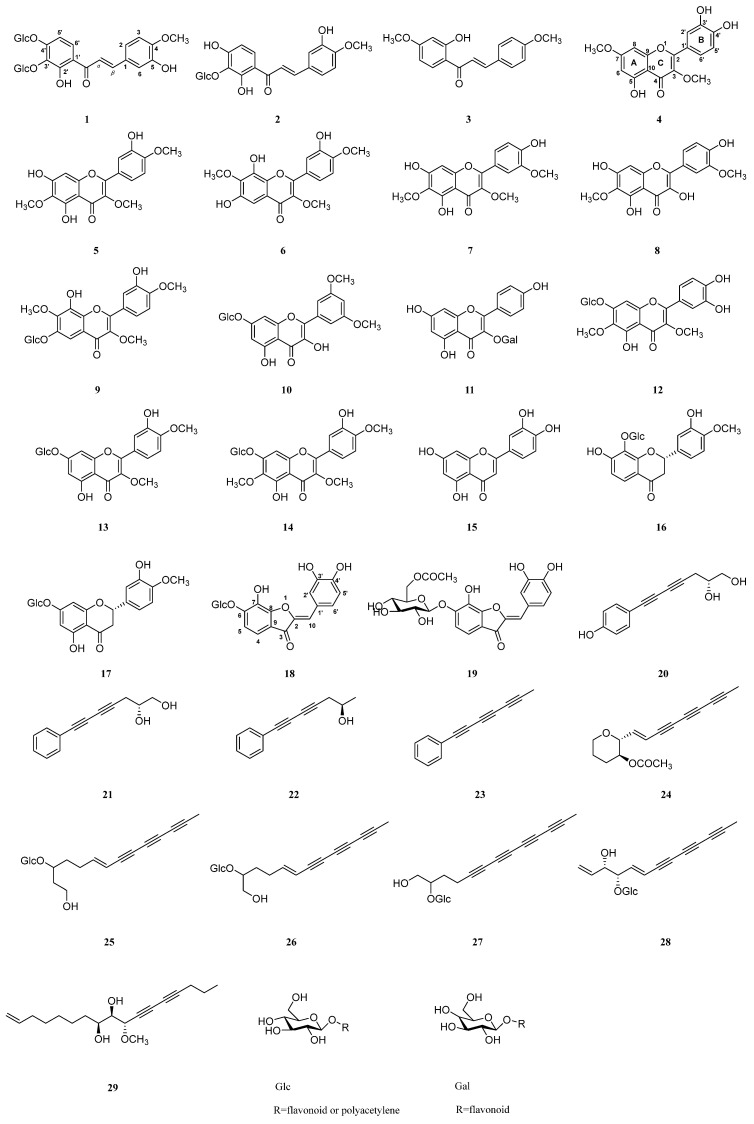
Chemical structures of compounds **1**–**29** isolated from *B. pilosa*.

In addition to **1** and **20**, eighteen flavonoids (**2**–**19**) [17,18,19,20,21,22,23,24,25,26,27,28,29,30,31,32,33,34] and nine polyacetylenes (**21**–**29**) [3,35,36,37,38,39,40,41,42] (Figure 3) were isolated from the methanol extract of the whole plant of *B. pilosa*; among them, compounds **8**, **11**, **17**, **28**, and **29** were isolated from this plant for the first time. LC-MS and 1D NMR were used to establish the structures of all the known compounds, and the spectra data for compounds **2**–**19** and **21**–**29** are described in the supporting information.

### 2.3. Compounds ***1**–**29*** Showed Cytotoxicities and Topo I Inhibitory Activities

The cytotoxicities of **1**–**29** were tested by an SRB assay on a panel of five human cancer cell lines and one human hepatocyte cell line. The results from Table 3 showed that compounds **4**, **5**, **6**, and **7** exhibited potent cytotoxicities against A549, HCT116, and DLD-1 cells, and their IC_50_ values were at the same levels compared to those of CPT, while, in MDA-MB-231 and HepG2 cells, they are less active than CPT. Compared to **4**, **5**, **6**, and **7**, compounds **3**, **8**–**10**, **13**, **15**, **20**–**23**, and **25**–**28** exhibited weak cytotoxicities with IC_50_ values varying from 50.50 μM to over 200 μM in different cell lines. Compounds **1**, **2**, **11**, **12**, **14**, **16**–**19**, **24**, and **29** exhibited no cytotoxicity in all cell lines at the tested concentration of 200 μM. In general, all the compounds were less active in HL-7702 than in other cancer cell lines.

**Figure 4:**
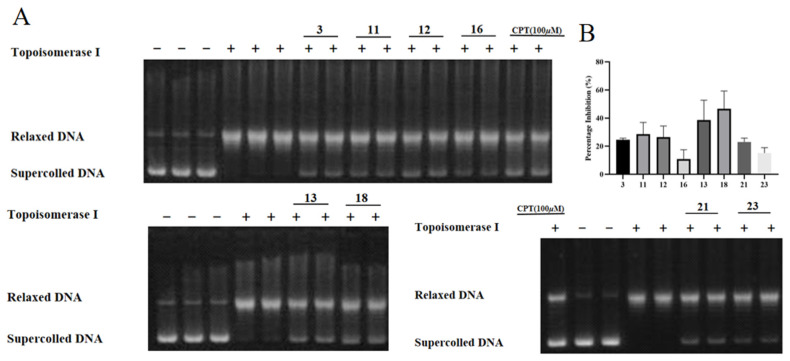
The inhibitory effect of flavonoids **3**, **11**, **12**, **16**, **13**, **18**, **21**, and **23** on DNA Topo I. (**A**) Representative images of the Topo I inhibition assay; the tested concentration of the flavonoids is 400 μM. (**B**) The percentage inhibition of the compounds on Topo I activity at the concentration of 400 μM.

A DNA relaxation assay was performed to determine the inhibitory effect of compounds **1**–**29** on Topo I. To remove false positive results, a preliminary assay of DNA cleavage was performed, and no cleavage effect was detected in any of the compounds (Figure S20). As the results show in Table 3, compounds **1**, **2**, **5**, **6**, **7**, **8**, **15**, **19**, **20**, **22**, and **24** were the active ones, with IC_50_ values of 393.5, 328.98, 145.57, 239.27, 224.38, 189.84, 89.91, 47.5, 301.32, 178.03, and 218.27 μM, respectively. As for the other compounds, though the IC_50_ values were over the maximum tested concentration, the percentage inhibition values of these compounds on Topo I activity were different at 400 μM. The results from Figure 4 show that **3**, **11**, **12**, **16**, **13**, **18**, **21**, and **23** exhibited inhibitory activities on Topo I at the concentration of 400 μM and the percentage inhibition values were 24.63%, 28.56%, 26.41%, 10.92%, 38.61%, 46.66%, 23.04%, and 15.18%, respectively, which indicated that these compounds showed weak inhibitory activity against DNA Topo I in vitro. For compounds **4**, **9**, **10**, **14**, **17**, and **25**~**29**, no inhibition at 400 μM was detected.

### 2.4. Compounds ***5**–**7*** Arrested Cell Cycle in Cancer Cells

**Figure 5:**
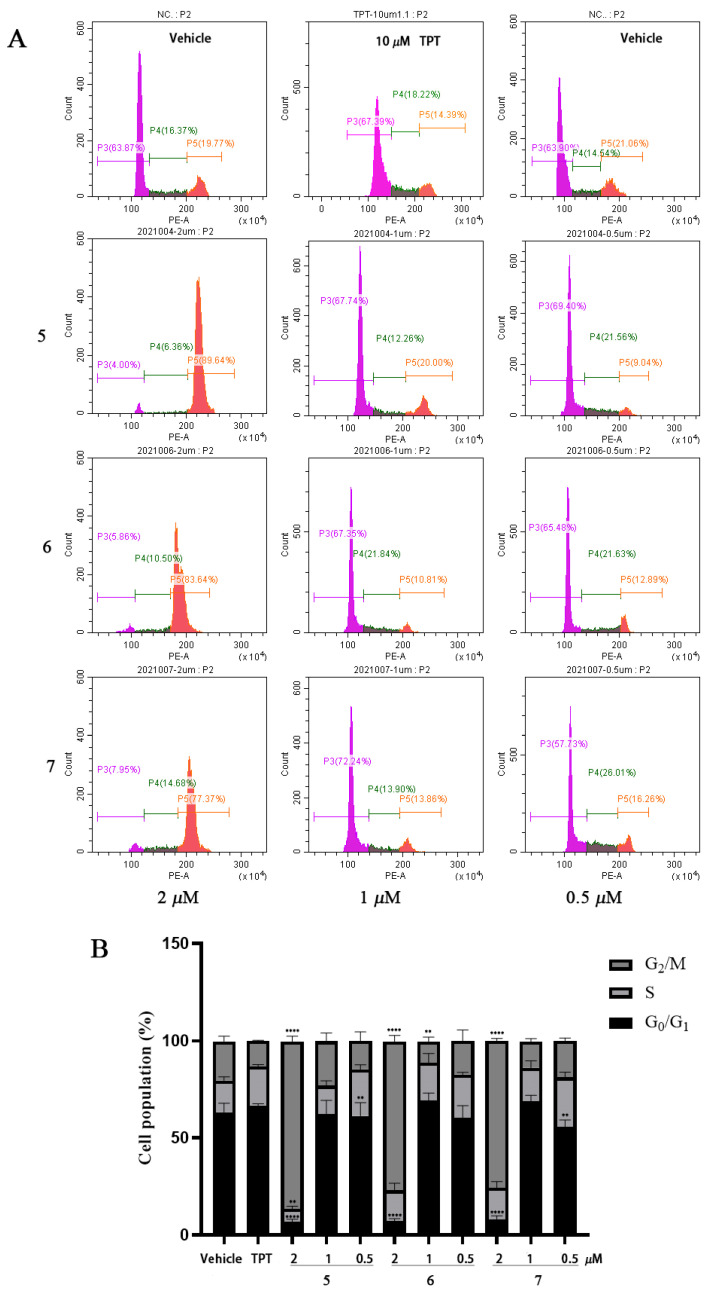
Results of flavonoid (**5**, **6**, and **7**)-induced cell cycle arrests in DLD-1 cells. (**A**) Representative images of flow cytometry analysis of the DNA content. (**B**) Statistic analysis of cells in the G_1_, S, and G_2_/M phases after treatment with **5**, **6**, and **7**. ** *p* < 0.01 and **** *p* < 0.0001 mean significant differences from the vehicle control. Data are expressed as mean ± SD values from three independent experiments.

The effect of flavonoids **5**, **6**, and **7** on the DLD-1 cell cycle was examined by DNA content analysis. The results show that all the compounds inhibited the cell growth in a cell cycle-nonspecific way, in which a potent G_2_/M-phase arrest was observed after treatment with a higher concentration, and the obvious cell cycle arrest in the G_1_ or S phase was monitored after treatment with a lower concentration. After treatment with 2 μM of compounds **5**, **6**, and **7** for 24 h, the percentages of the cell population in the G_2_/M phase significantly increased from 20.12% to 86.05%, 76.49%, and 75.75%, and the cells in the G_1_/S (combined) phase remarkably decreased from 79.53% to 13.46%, 22.91%, and 24.26%, respectively, which means a potent G_2_/M cell cycle arrest caused by the treatment (*p* < 0.0001). In addition, after treatment with 1 and 0.5 μM of compounds **5**, **6**, and **7** for 24 h, the percentages of cells in the G_1_/S phase increased from 79.53% to 76.83%, 88.65% (*p* < 0.0001), and 86.07% (*p* < 0.001) and from 79.53% to 85.30% (*p* < 0.01), 82.61%, and 81.16% (*p* < 0.01), respectively, which indicates that an apparent G_1_- or S-phase arrest was induced after the treatment with a lower compound concentration (Figure 5).

### 2.5. Compound ***5*** Regulated Cell Cycle-Related Protein Expression in Cancer Cells

**Figure 6:**
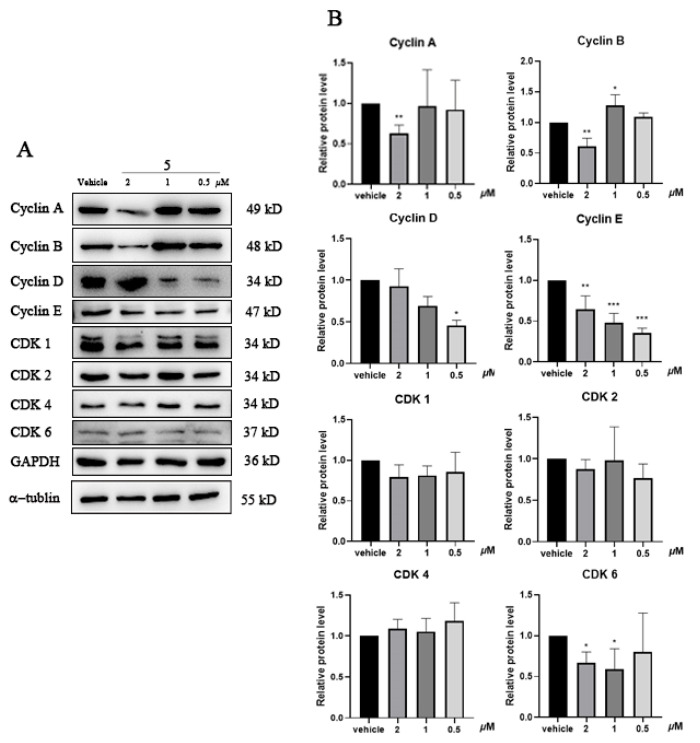
Effects of flavonoid **5** on cell cycle-relevant proteins in DLD-1 cells. (**A**) Representative immunoblots of the protein expression. (**B**) Histograms for the quantified results of protein levels, which were adjusted with corresponding α-tubulin/GAPDH protein levels and expressed as folds of the control. Experiments were independently repeated at least three times. * *p* < 0.05, ** *p* < 0.01 and *** *p* < 0.001 mean significant differences from the vehicle group.

As a compound that showed potent inhibitory activity on DNA Topo I and cytotoxicity on cancer cell lines, the effect of **5** on cell cycle-related protein expression levels was investigated by Western blot in DLD-1 cells after treatment for 24 h. The results show that compound **5** potently decreased the expression levels of cyclin A, cyclin B, and CDK6 at 2 μM; meanwhile, **5** caused a potent downregulation of cylcin D, cyclin E, and CDK6 at 1 and 0.5 μM (Figure 6). The results indicate that, at different concentration of the inhibitor treated, flavonoid **5** caused G_1_/S- or G_2_/M-phase cell cycle arrests by regulating the expression of different cell cycle-related proteins.

## 3. Materials and Methods

### 3.1. Plant Material

The samples of *B. pilosa* L. were collected from the Liangwangshan mountain, Kunming, Yunnan province, China, in June 2020 and authenticated by Kunming Plant Branch Biotechnology Co., Ltd. (Zhang Jun) (Kunming, China), and a voucher specimen (YMU-ZF20200624) was deposited at the Key Laboratory of Chemistry in Ethnic Medicinal Resources, State Ethnic Affairs Commission and Ministry of Education, Yunnan Minzu University.

### 3.2. General Experimental Procedures

NMR experiments were conducted on a Bruker DRX-400 spectrometer operating at 400 MHz (^1^H) and 100 MHz (^13^C) at 300 K (chemical shifts *δ* in ppm, coupling constants *J* in Hz) (Bruker, Ettlingen, Germany). LC-MS data were obtained with an Agilent liquid chromatography-G6400 series triple quadrupole mass spectrometer (Agilent Technologies, Santa Clara, CA, USA). High-performance liquid chromatography (HPLC) separation was performed on an Agilent 1260 series with Agilent ZORBAX SB (21.2 × 250 mm) preparative column packed with C18 (7 µm) (Agilent Technologies, Santa Clara, CA, USA), Agilent ZORBAX XDB (9.4 × 250 mm) semi-preparative column packed with C18 (5 µm), and Agilent ZORBAX XDB (4.6 × 250 mm) analytical columns packed with C18 (5 µm). The thin-layer chromatography (TLC) analysis were performed with Merck silica gel 60 GF254 aluminum sheet (Merck, Darmstadt, Germany), and the spots were first viewed under UV light at *λ* 254 nm and 365 nm, and then stained with benzaldehyde (prepared from 135 mL ethanol + 5 mL sulfuric acid + 1.5 mL acetic acid + 3.7 mL CH_3_OC_6_H_4_CHO) followed by heating. Column chromatography (CC) was performed on Sephadex LH-20 (GE Healthcare, Waukesha, WI, USA), silica gel (100, 200, 200–300, or 300–400 mesh) (Qingdao Marine Chemical Inc., Qingdao, China), C18 reversed-phase silica gel (S-50 μm) (YMC, Kyoto, Japan), and MCI GEL (CHP20/P120) (Mitsubishi Chemical, Tokyo, Japan). All solvents used for chromatographic separations were distilled before use.

### 3.3. Extraction and Isolation

Powdered and air-dried whole plant of *B. pilosa* (20 kg) were extracted 7 times with 95% methanol (MeOH) by maceration for 24 h at room temperature. The MeOH extracts (2 kg) were combined and concentrated under reduced pressure. A portion of this extract was suspended in water (H_2_O) and successively extracted with light petroleum (PE), ethyl acetate (EtOAc), and *n*-butanol (*n*-BuOH) to obtain PE (310 g), EtOAc (165 g), and *n*-BuOH (360 g) fractions, respectively. The PE extract (310 g) was fractionated by CC (2500 g) on silica gel (60–100 mesh, 15 cm × 100 cm) and eluted with PE/DCM (100:1 to 1:1, *v*/*v*), PE/EtOAc (5:1 to 1:1, *v*/*v*) to obtain 8 fractions (A-H). Fr.A (1.3 g) was analyzed using TLC, and the same components were merged to obtain 3 fractions (FrA.1-FrA.3). FrA.1 (810.2 mg) was separated using silica gel CC (100–200 mesh) and eluted with PE/DCM (100:1 to 1:1, *v*/*v*) to yield 3 fractions (FrA.1.1-FrA.1.3); FrA.1.1 was further separated using silica gel CC (200–300 mesh) and eluted with PE/DCM (100:1, *v*/*v*) and Sephadex LH-20 column (MeOH) to yield compound **3** (50.6 mg). FrA.1.2 was separated using Sephadex LH-20 column (DCM:MeOH = 1:1) and silica gel to obtain compound **23** (32.4 mg). FrA.1.3 was fractionated by Sephadex LH-20 column (DCM:MeOH = 1:1) and silica gel to obtain compound **22** (20.1 mg). FrA.2 was further separated by silica gel CC, eluted with PE/EtOAc (100:1, *v*/*v*) and HPTLC, and eluted with PE/EtOAc (15:1, *v*/*v*) to obtain compound **24** (12.3 mg). The EtOAc extract (165 g) was fractionated by CC (2500 g) on silica gel (60–100 mesh, 15 × 100 cm) and eluted with PE and DCM/MeOH (1:0 to 0:1, *v*/*v*) to obtain 7 fractions (Fr.I-Fr.O). Fr.K (DCM: MeOH = 50:1) was analyzed using TLC, and the same components were merged to obtain 6 fractions (FrK.1-FrK.6). FrK.1 was subjected to middle chromatogram isolated (MCI) gel and eluted with MeOH/H_2_O (50:50 to 1:0, *v*/*v*) to yield 7 fractions (FrK.1.1-FrK.1.7); FrK.1.4 was fractionated by Sephadex LH-20 column (MeOH), silica gel, and ODS-C18 column and eluted with MeOH/H_2_O (40:60 to 1:0, *v*/*v*) to yield compound **21** (25 mg). FrK.1.6 was separated using a Sephadex LH-20 column (MeOH) to obtain 3 fractions (FrK.1.6.1-FrK.1.6.3); FrK.1.6.2 was further separated using silica gel CC (200–300 mesh), eluted with DCM/MeOH (1:0 to 0:1, *v*/*v*), and then depurated by Sephadex LH-20 column (MeOH) to obtain compound **4** (25 mg) and compound **5** (30 mg). FrK.1.7 was separated using a Sephadex LH-20 column (MeOH) to obtain 4 fractions (FrK.1.7.1-FrK.1.7.4); FrK.1.7.3 was subjected to the ODS-C18 column and eluted with MeOH/H_2_O (30:70 to 1:0, *v*/*v*) to yield compounds **6** (15 mg) and **7** (15 mg). Fr.L (DCM: MeOH = 25:1) was analyzed using TLC and 3 fractions were obtained (FrL.1-FrL.3); FrL.2 was depurated by MCI, silica gel, Sephadex LH-20 (MeOH), and C18 column and eluted with MeOH/H_2_O (30:70 to 1:0, *v*/*v*) to yield compound **20** (10 mg). Fr.M (DCM: MeOH = 10:1) was analyzed using TLC, and the same components were merged to obtain 6 fractions (FrM.1-FrM.6); FrM.6 was depurated by MCI and eluted with MeOH/H_2_O (50:50 to 1:0, *v*/*v*) to yield 3 fractions (FrM.6.1-FrM.6.3). FrM.6.1 was separated using a Sephadex LH-20 column (MeOH) to obtain 6 fractions (Fr.6.1.1-Fr.6.1.6); Fr.6.1.3 was fractionated by reverse-phase chromatography (RP-18) and then purified with chromatography silica gel and Sephadex LH-20 (MeOH) to obtain compounds **25** (5 mg) **27** (3.5 mg), **28** (7.1 mg), and **29** (10.4 mg). Fr.6.1.4 was depurated by repeated preparative HPLC and eluted with MeOH/H_2_O (70:30, *v*/*v*, ν = 4 mL/min) to obtain 4 fractions (FrM.6.1.4.1-FrM.6.1.4.4). FrM.6.1.4.2 was further separated by recrystallization to obtain compound **9** (15 mg). FrM.6.1.4.1 was fractionated by RP-18 and then purified with chromatography silica gel, Sephadex LH-20 (MeOH), iterative semi-preparative HPLC, and recrystallization to obtain compounds **1** (13 mg), **10** (18 mg), **11** (18 mg), **12** (20 mg), **13** (15 mg), **16** (9 mg), and **17** (4 mg). FrM.6.1.4.3 was separated by iterative semi-preparative HPLC to obtain compound **26** (50 mg). Fr.6.1.6 was depurated by LH-20 and iterative semi-preparative HPLC and eluted with MeOH/H_2_O (50:50, *v*/*v*, ν = 1.5 mL/min) to obtain compound **15** (3 mg). Fr.6.1.5 was further separated by repeated preparative HPLC and eluted with MeOH/H_2_O (53:47, *v*/*v*, ν = 4 mL/min), iterative semi-preparative HPLC and eluted with ACN/H_2_O (19:81, *v*/*v*, ν = 2.5 mL/min), MeOH/H_2_O (37:63, *v*/*v*, ν = 2 mL/min), and recrystallization to obtain compounds **2** (22 mg), **8** (7 mg), **18** (25 mg), and **19** (2.8 mg). The *n*-BuOH extract (360 g) was fractionated by macroporous absorption resin and eluted with EtOH/H_2_O (50:50 to 1:0, *v*/*v*) to yield 3 fractions (Fr.P-Fr.R). Fr.P was separated using a Sephadex LH-20 column (MeOH), then depurated by repeated preparative HPLC, and eluted with MeOH/H_2_O (70:30, *v*/*v*, ν = 4 mL/min) to obtain 4 fractions (Fr.P.1-Fr.P.4). Fr.P.3 was further separated using semi-preparative HPLC and eluted with MeOH/H_2_O (42:58, *v*/*v*, ν = 2 mL/min) to obtain compound **14** (7 mg).

### 3.4. Cell Culture

The cell lines MDA-MB-231 (No. TCHu227), A549 (No. TCHu150), and HepG2 (No. TCHu72) were obtained from the Cell Bank/Stem Cell Bank, Chinese Academy of Sciences (Shanghai, China). The cell lines DLD-1 (No. CC0507) and HCT116 (No. CC0506) were obtained from the Cell Bank of Cellcook Biotech Co., Ltd. (Guangzhou, China). Cell line HL-7702 (No. BNCC338358) was obtained from Bena Culture Collection (Zhengzhou, China).

The human cancer cell lines MDA-MB-231 (breast adenocarcinoma), DLD-1 (colorectal adenocarcinoma), A549 (lung epithelial carcinoma), HCT116 (colorectal adenocarcinoma), HepG2 (hepatocellular carcinoma), and human hepatocyte HL-7702 were cultured in Dulbecco’s modified eagle medium (DMEM, Biological Industries, Beit Haemek, Israel) containing 10% (*v*/*v*) fetal bovine serum (FBS, Biological Industries, Beit Haemek, Israel) at 37 °C in a humidified incubator (ThermoFisher, Carlsbad, CA, USA) with a 5% CO_2_ atmosphere.

### 3.5. Cytotoxicity Assay

The cytotoxic activities of the compounds against cancer cells were examined with a sulforhodamine B (SRB) colorimetric assay. Cells were seeded at a density of 5 × 10^3^/well in 96-well plates for 24 h; various concentrations of the tested compounds, dissolved in DMSO and diluted with the complete medium, were added in the wells in triplicate and incubated for 48 h. After that, the cell viability was assessed with the SRB assay as we described before [43]. The IC_50_ values were determined using the Reed and Muench method and are expressed as the mean ± SD of at least three independent measurements. Camptothecin (CPT) (Energy Chemical, Shanghai, China) was used as a reference compound for the positive control.

### 3.6. Topo I Inhibition Assay

The DNA relaxation assay was adopted to investigate the compounds’ inhibitory activities on Topo I. Different concentrations of the compounds, dissolved and diluted in DMSO, were added into the relaxation buffer (50 mM Tris, pH 7.5, 50 mM KCl, 10 mM MgCl_2_, 0.1 mM EDTA, 0.5 Mm dithiothreitol, and 0.05 mg/mL bovine serum albumin) containing 0.2 U Topo I (ThermoFisher, Waltham, MA, USA) and incubated at room temperature for 10 min. After that, 500 ng supercoiled pBR322 plasmid DNA (ThermoFisher, Waltham, MA, USA) was added and then incubated at 37 °C for another 30 min. Finally, the reactions were stopped with a stopping solution (5% sodium dodecyl sulfate, 0.0025% bromophenol blue, and 25% glycerol), and the mixtures were applied onto 0.8% agarose gel and subjected to electrophoresis for 1.5 h in Tris-Acetate-EDTA buffer. After being stained with GelRed (Biotium, Fremont, CA, USA), the gel was imagined with the imaging system (Biotop, Shanghai, China). CPT (Energy Chemical, Shanghai, China) was used as a reference compound for the positive control. The band density of the supercoiled DNA was quantified by the ImageJ software (version 1.53a, National Institutes of Health, Bethesda, MD, USA), and the inhibition activity is expressed as the percentage inhibition and calculated by formula: Inhibition % = [(A − B)/(C − B)] × 100%, where A is the band density of the tested samples, B is the band density of the negative control, and C is the band density of the blank control. IC_50_ values were determined using the Reed and Muench method and are expressed as the mean ± SD of at least three independent measurements in duplicate.

### 3.7. DNA Content Analysis

Cells were seeded in 6-well plates and allowed to adhere for 24 h at 37 °C. After treatment with different concentrations of the samples for another 24 h, the cells were harvested and washed with PBS, and then, the DNA content analysis was conducted with a cell cycle staining kit (Multi sciences, Hangzhou, China), according to the manufacturer’s instructions. The fluorescence intensity of the cells was measured by flow cytometry (Beckman CytoFlex, Miami, FL, USA). Topotecan (TPT) (Solarbio, Beijing, China) was used as a reference compound for the positive control.

### 3.8. Western Blot Analysis

Cells treated with the compounds were harvested, and the total protein was isolated with lysis buffer (72 mM Tris pH 6.8, 2% SDS, 10% glycerol, and 0.1% bromophenol blue). The proteins were subjected to SDS-PAGE and transferred to a 0.45 μm PVDF membrane (Millipore, Burlington, USA). After being blocked with 5% non-fat milk in TBST, the membranes were washed and then incubated with specific primary antibodies (Cdk2 and Cdk6 were purchased from BBI Life Sciences Corporation, Shanghai, China; cyclin A, cyclin B, cyclin D, cyclin E, Cdk1, and Cdk4 were purchased from Proteintech, Rosemont, IL, USA) in TBST containing 2% non-fat milk overnight at 4 °C. After washing, the membranes were incubated with secondary horseradish peroxidase (HRP)-conjugated antibodies at room temperature for 1 h. The blot was visualized using an enhanced chemiluminescence assay kit (Meilunbio, Dalian, China) under a chemiluminescence detector (Tanon, Shanghai, China). The band density of the blot was quantified by the ImageJ software with at least three independent experiments.

### 3.9. Statistical Analysis

All the results were calculated by at least three independent experiments and are presented as mean ± S.D. values. A one-way ANOVA or the Student’s *t*-test was used for the statistical analysis. A *p* value < 0.05 was considered statistically significant.

## 4. Discussion

Cancer is commonly characterized by uncontrolled cell division and proliferation, which are directly related to DNA replication. Targeting Topos disrupts DNA replication and transcription, which leads to the inhibition of cell division, thereby stopping the growth of cancer cells [12]. In a wide range of human solid tumors, the intracellular level of Topo I is higher than that in normal tissues, especially in metastatic ovarian cancer, cervical cancer, small-cell lung cancer, metastatic colon cancer, and pancreatic cancer [11], suggesting that controlling the Topo I level is essential in treating cancers. Several studies have revealed that specific flavonoids in natural products inhibit the activity of DNA Topo I [44]. Licochalcones A, a chalcone isolated from *Glycyrrhiza inflata*, and luteolin, a flavone isolated from the leaves of *Vitex negundo*, were reported to show cytotoxicity by inhibiting DNA Topo I [45,46]; therefore, flavonoids hold therapeutic prospects in the treatment of cancers.

*B. pilosa* L. is a herb that is used as a folk medicine for cancer treatment in China. In this study, nineteen flavonoids and ten polyacetylenes (Figure 3) were isolated from the plant, including one flavone (**15**), eleven flavonols (**4**–**14**), two flavanones (**16**–**17**), three chalcones (**1**–**3**), two aurone (**18**, **19**), and ten polyacetylenes (**20**–**29**), and their inhibitory activities on DNA Topo I and cytotoxicities on cancer cell lines were tested (Table 3). The new compounds **1** and **20** and the polyacetylenes **22** and **24** were found to be the inhibitors of DNA Topo I for the first time. The structure–activity relationship of flavonoids against Topo I was analyzed. Based on the results from Table 3 and Figure 4, it was demonstrated that, compared to chalcones, flavones, flavonols, and aurones, flavonoids showed a strong inhibitory activity against DNA Topo I in vitro. Compared to flavanones, flavones and flavonols are more active, indicating that the double bond between C2 and C3 is important to the inhibitory activity, which was verified by the observation of the decreased inhibitory activities of flavanones (**16** and **17**), which lack the C2-C3 unsaturation bond on the C-ring. By comparing the activity of **4** with those of **5**, **6**, and **7**, we deduced that the substitution of the C-6 position on the A-ring of flavonols was essential for Topo I inhibition, and if there was no substitution, the activity potently decreased. Additionally, if the substitution at the C-6 position was glycosylated, the activities of the compounds also decreased, which was verified by comparing **5**, **6**, **7**, and **8** with **9**. The C-7 position is also important for the inhibitory activity, which was enhanced by the substitution of small groups (-OH and -OCH_3_), and if the substitution was glycosylated, the activities of the compounds (**10**, **12**, **13**, **14**, and **17**) potently decreased, which was also confirmed by comparing **14** with its aglycone **5**. As for the B-ring of flavonols, substitutions at C-3′ and C-4′ are important for the inhibitory activity (**5**, **6**, and **7**), and comparing **5** with **7**, the activity of the hydroxy substitution at the C-3′ position is better than that of the methoxy substitution. Furthermore, the inhibition results from compounds **12** and **14** indicates that the hydroxyl group substitution at C-3′ and C-4′ is superior to that of methoxy group, which is correlated with what has been described in previous publications [47,48]. As for the cytotoxicity of compounds, only **5**, **6**, **7**, **15**, and **22** showed good activity in cancer cell lines, as well as in the DNA Topo I assay, which indicates that their cytotoxicity was partly caused by DNA Topo I inhibition. As for compound **4**, though inactive against Topo I, a good cytotoxicity on cancer cells was observed (not as good as those of **5**, **6**, and **7**), indicating that DNA Topo I is only one of the cytotoxic targets of flavonoids, which intervenes in the characteristics of multi-targets for natural products [44]. As the most active inhibitor of Topo I, **15**, a more hydrophilic compound compared to **5**, **6**, and **7**, shows a weaker cytotoxicity, which is attributed to the lipid bilayer of cell membranes that prevents the entrance of **15** into the cytosol, leading to less cytotoxic activity. Comparing aurones **18** and **19**, the esterification of the glucose of **19** enhanced the inhibitory effect on Topo I. Although **19** showed a good inhibitory activity, its cytotoxicity was weak, which may be attributed to the metabolized difference between cellular experiments and non-cellular ones.

Topoisomerase I-targeting drugs exert a cytotoxic effect by producing enzyme-mediated DNA damage, which leads to cell cycle arrest. Thus, the cell cycle analysis of DLD-1 cells treated with **5**, **6**, and **7** was conducted by flow cytometry. The results from the DNA content (Figure 5) analysis show that all flavonoids caused potent G_2_/M arrests at a higher concentration and significant G_1_/S arrests at a lower concentration, which indicates that flavonoids **5**, **6**, and **7** induced double-strand DNA breaks by the inhibition of DNA Topo I; then, the activated DNA damage response accumulated cells in different phases depending on the concentration of the inhibitor used. The cell cycle-nonspecific arrest by flavonoids is in agreement with what was reported for DNA damaging agents in previous publications [49,50]. The eucaryotic cell cycle is regulated by the periodic synthesis and destruction of cyclins that complex with cyclin-dependent kinases (Cdks); thus, cyclin/Cdk complexes are the key regulators for governing the checkpoints in a cell division cycle. In order to verify the results of the DNA content analysis, the levels of different cyclins and Cdks in the cell cycle were investigated by Western blot (Figure 6). The results exhibit that compound **5** caused cell cycle arrest by downregulating the expression of different cyclins or Cdks at different concentrations. As the main regulator for controlling the G_2_/M checkpoint, the cyclin B/Cdk1 complex is the key regulator of cell mitosis, and treatment with 2 μM of compound **5** caused a significant decrease in cyclin B, and because the periodic synthesis and destruction of cyclins control the activation of the Cdks and the cyclin/Cdk complexes [51], it suggests that the reduction in cyclin B induced by **5** blocked the cells’ entry into mitosis and led to the potent G_2_/M arrest in DLD-1 cells. In the normal eucaryotic cell cycle process, the activity of the cyclin D/Cdk4/6 and cyclin E/Cdk2 complexes controls G_1_-phase progression, and the cyclin A/Cdk2 complex controls the S phase. In this study, the results from the Western blot show that the treatment with lower concentrations of compound **5** decreased the expression of cyclin D, cyclin E, and Cdk6 (Figure 6), which indicates that **5** inhibits the activity of the cyclin D/Cdk4/6, cyclin A/Cdk2, and cyclin E/Cdk2 complexes at a lower concentration and arrests the G_1_/S cell cycle checkpoint in DLD-1 cells. The results from the Western blot are consistent with that from the DNA content analysis. Additionally, as it was shown in DLD-1 cells, compound **5** also caused potent G_2_/M arrests at 2 μM and significant G_1_/S arrests at 0.5 μM and regulated the expression of cell cycle-related proteins in the HCT116 cell line (Figure S21). All the results indicate that the flavonoids and polyacetylenes that exist in *B. pilosa* are the cytotoxic components responsible for its anti-tumor activity by inhibiting DNA Topo I.

## 5. Conclusions

Flavonoids broadly exist in plants and exhibit a wide range of bioactivities. *B. Pilosa* is a traditional herb that is used as anti-tumor folk medicine in China and many other countries. However, its main constituents with anti-tumor activities have not been identified. In this study, a new chalcone and a new polyacetylene, together with twenty-seven known flavonoids and polyacetylenes, were isolated from *B. pilosa*, and their inhibitory activities against DNA Topo I were reported for the first time, except for compound **15**, which was isolated from the leaves of *Vitex negundo* L. and has been reported to show good activity [46]. The study of the structure–activity relationship analysis and cytotoxicity mechanism indicated that Topo I-targeting flavonoids and polyacetylenes are two kinds of active ingredients that lead to the anti-tumor effect of *B. pilosa*, which is of great importance for the R&D of flavonoids and the traditional herb *B. pilosa*.

## Figures and Tables

**Table 1:** ^1^H NMR and ^13^C NMR of compound **1** (DMSO-*d*_6_, *δ*ppm).

No.	^13^C NMR (*δ* ppm)	^1^H NMR (*δ* ppm)	No.	^13^C NMR (*δ* ppm)	^1^H NMR (*δ* ppm)
C=O	192.1		1″	103.8	4.85 (1H, d, *J* = 7.6)
*α*	119.3	7.77 (1H, d, *J* = 15.4)	2″	74.1	3.29 (1H, overlapped)
*β*	144.9	7.70 (1H, d, *J* = 15.4)	3″	76.3	3.22 (1H, overlapped)
1	127.5	-	4″	69.7	3.13 (1H, overlapped)
2	115.1	7.34 (1H, d, *J* = 1.9)	5″	77.2	3.10 (1H, overlapped)
3	146.7	-	6″	60.8	3.44 (1H, overlapped) 3.62 (1H, m)
4	150.6	-	1‴	100.7	4.98 (1H, d, *J* = 7.4)
5	112.0	7.01 (1H, d, *J* = 8.4)	2‴	73.4	3.35 (1H, overlapped)
6	122.6	7.32 (1H, dd, *J* = 8.4, 1.9)	3‴	76.0	3.30 (1H, overlapped)
1′	116.8	-	4‴	69.8	3.17 (1H, overlapped)
2′	156.5	-	5‴	77.4	3.40 (1H, overlapped)
3′	133.6	-	6‴	60.7	3.47 (1H, overlapped) 3.71 (1H, m)
4′	155.6	-	4-OCH_3_	55.8	3.84 (3H, s)
5′	106.9	6.84 (1H, d, *J* = 9.3)	3-OH		9.25 (1H, s)
6′	127.1	8.02 (1H, d, *J* = 9.3)	2′-OH		12.90 (1H, br s)

**Table 2:** ^1^H NMR and ^13^C NMR of compound **20** (CD_3_OD, *δ* ppm).

No.	^13^C NMR (*δ* ppm)	^1^H NMR (*δ* ppm)	No.	^13^C NMR (*δ* ppm)	^1^H NMR (*δ* ppm)
1	66.0	3.54 (1H, dd, *J* = 11.2, 5.8) 3.59 (1H, dd, *J* = 11.2, 4.9)	1′	113.5	-
2	71.7	3.77 (1H, dddd, *J* = 6.2, 6.0, 5.8, 4.9)	2′, 6′	135.1	7.28 (2H, d, *J* = 8.6)
3	25.2	2.50 (1H, dd, *J* = 17.3, 6.2) 2.60 (1H, dd, *J* = 17.3, 6.0)	3′, 5′	116.6	6.72 (2H, d, *J* = 8.6)
4	80.8	-	4′	160.0	-
5	67.6	-			
6	73.2	-			
7	76.2				

**Table 3:** The cytotoxicities against five cancer cell lines and Topo I inhibitory activities of compounds **1**–**29**.

Comp.	IC_50_ (μM)						
	Cytotoxicity						Topo I
	A549	HCT116	MDA-MB-231	HepG2	DLD-1	HL-7702	
**1**	>200	>200	>200	>200	>200	>200	393.50 ± 38.99
**2**	>200	>200	>200	>200	>200	>200	328.98 ± 58.77
**3**	135.56 ± 25.04	177.96 ± 6.11	166.71 ± 5.63	>200	117.49 ± 11.60	>200	>400
**4**	8.16 ± 4.04	4.52 ± 0.04	>200	3.05 ± 1.31	7.14 ± 0.35	19.61 ± 0.62	>400
**5**	2.16 ± 0.30	0.79 ± 0.13	39.67 ± 4.58	40.3 ± 11.14	0.60 ± 0.05	14.36 ± 0.45	145.57 ± 7.88
**6**	3.09 ± 0.28	0.59 ± 0.10	>200	34.59 ± 8.01	1.58 ± 0.07	23.91 ± 5.73	239.27 ± 31.35
**7**	0.86 ± 0.03	0.43 ± 0.03	81.57 ± 7.43	28.38 ± 13.05	0.85 ± 0.02	11.44 ± 4.42	224.38 ± 27.18
**8**	177.84 ± 4.38	>200	>200	>200	186.79 ± 48.65	>200	189.84 ± 22.09
**9**	135.31 ± 32.29	87.68 ± 15.27	>200	>200	50.50 ± 0.31	125.54 ± 7.08	>400
**10**	>200	59.29 ± 4.56	>200	>200	63.00 ± 4.08	114.75 ± 0.55	>400
**11**	>200	>200	>200	>200	>200	>200	>400
**12**	>200	>200	>200	>200	>200	>200	>400
**13**	>200	139.10 ± 11.31	>200	>200	178.68 ± 5.94	>200	>400
**14**	>200	>200	>200	>200	>200	>200	>400
**15**	93.92 ± 2.92	31.70 ± 1.54	94.91 ± 0.50	174.80 ± 16.64	69.17 ± 4.04	>200	89.91 ± 28.08
**16**	>200	>200	>200	>200	>200	>200	>400
**17**	>200	>200	>200	>200	>200	>200	>400
**18**	>200	>200	>200	>200	>200	>200	>400
**19**	>200	>200	>200	>200	>200	>200	47.50 ± 15.3
**20**	194.01 ± 12.77	173.81 ± 2.47	>200	>200	>200	>200	301.32 ± 34.94
**21**	186.68 ± 6.63	>200	>200	>200	>200	>200	>400
**22**	124.84 ± 23.42	117.63 ± 5.70	146.00 ± 7.17	178.53 ± 3.62	141.56 ± 10.15	160.56 ± 2.18	178.03 ± 45.72
**23**	129.78 ± 4.93	70.91 ± 3.73	139.89 ± 5.53	167.79 ± 10.78	122.54 ± 8.76	198.81 ± 0.86	>400
**24**	>200	>200	>200	>200	>200	>200	218.27 ± 50.82
**25**	173.81 ± 12.3	162.38 ± 15.77	>200	>200	187.21 ± 17.4	>200	>400
**26**	187.84 ± 15.83	>200	>200	>200	>200	>200	>400
**27**	187.87 ± 14.88	112.23 ± 6.59	164.29 ± 11.74	>200	144.41 ± 7.36	>200	>400
**28**	190.39 ± 12.09	>200	>200	>200	>200	>200	>400
**29**	>200	>200	>200	>200	>200	>200	>400
**^a^ CPT**	4.41 ± 1.11	12.07 ± 5.74	20.41 ± 3.70	1.71 ± 0.47	16.96 ± 3.82	2.11 + 2.05	11.11 ± 3.43

^a^ the reference compounds of the positive control.

## Data Availability

The original contributions presented in the study are included in the article (and Supplementary Material), further inquiries can be directed to the corresponding authors.

## Supplementary Materials

The following supporting information can be downloaded at: https://www.mdpi.com/article/10.3390/molecules29153547/s1. Figure S1: HRESI-MS spectrum of compound **1**. Figure S2: ^1^H-NMR (DMSO-*d*_6_) spectrum of compound **1**. Figure S3: ^13^C-NMR (DMSO-*d*_6_) spectrum of compound **1**. Figure S4: DEPT (90°, 135°) spectrum of compound **1**. Figure S5: HSQC (DMSO-*d*_6_) spectrum of compound **1**. Figure S6: HMBC (DMSO-*d*_6_) spectrum of compound **1**. Figure S7: ^1^H-^1^H COSY (DMSO-*d*_6_) spectrum of compound **1**. Figure S8: ROESY (DMSO-*d*_6_) spectrum of compound **1**. Figure S9: UV spectrum of compound **1**. Figure S10: IR spectrum of compound **1**. Figure S11: HRESI-MS spectrum of compound **20**. Figure S12: ^1^H-NMR (CD_3_OD) spectrum of compound **20**. Figure S13: ^13^C-NMR (CD_3_OD) spectrum of compound **20**. Figure S14: DEPT (90°,135°) spectrum of compound **20.** Figure S15: HSQC (CD_3_OD) spectrum of compound **20**. Figure S16: HMBC (CD_3_OD) spectrum of compound **20**. Figure S17: ^1^H-^1^H COSY (CD_3_OD) spectrum of compound **20**. Figure S18: UV spectrum of compound **20**. Figure S19: IR spectrum of compound **20**. Figure S20: The effects of compounds on DNA cleavage. Figure S21: Results of flavonoid **5** induced cell cycle arrests in HCT116 cells. Characterization data of compounds **2**–**19** and **21**–**29**.
