# Supplementary material for: Inhibition of DNA Topoisomerase Ι by Flavonoids and Polyacetylenes Isolated from Bidens pilosa L
Source: Molecules. 2024 Jul 27;29(15):3547. doi: 10.3390/molecules29153547 (PMC11314063; doi:10.3390/molecules29153547)
Supplement: Supplementary file 1 [file molecules-29-03547-s001.zip › molecules-3080299-supplementary.pdf]

SUPPORTING INFORMATION

Inhibition of DNA Topoisomerase I by Flavonoids and Polyacetylenes Isolated from *Bidens pilosa* L.

Guiyuan Zeng [1,2,†], Yinyue Wang [1,2,†], Meihua Zhu [1,2], Jumei Yi [1,2], Junjie Ma [1,2], Bijuan Yang [1,2], Weiqing Sun [1,2], Fang Dai [3], Junlin Yin [1,2,*] and Guangzhi Zeng [1,2,*]

[1] Key Laboratory of Chemistry in Ethnic Medicinal Resources, State Ethnic Affairs Commission and Ministry of Education, Yunnan Minzu University, Kunming 650504, China; yuan8556@126.com (G.Z.); wyy13995611885@163.com (Y.W.); zhm1773@163.com (M.Z.); 18468119534@163.com (J.Y.); mjj1336042031@163.com (J.M.); bijuan051322@163.com (B.Y.); weiqingsun1985@163.com (W.S.)

[2] Yunnan Key Laboratory of Chiral Functional Substance Research and Application, Yunnan Minzu University, Kunming 650504, China

[3] School of Chemistry and Environmental Engineering, Qujing Normal University, Qujing 655011, China; daifangld@163.com

* Correspondence: yinjunlin1979@sina.com (J.Y.); g.zh_zeng@ymu.edu.cn (G.Z.)

† These authors contributed equally to this work.

Supporting Information List

Figure S1. HRESI-MS spectrum of compound **1**……………………………………3

Figure S2. ^{1}H-NMR (DMSO-d_6) spectrum of compound **1**………………………...4

Figure S3. ^{13}C-NMR (DMSO-d_6) spectrum of compound **1**……………………...5

Figure S4. DEPT (90°, 135°) spectrum of compound **1**……………………………..6

Figure S5. HSQC (DMSO-d_6) spectrum of compound **1**……………………………7

Figure S6. HMBC (DMSO-d_6) spectrum of compound **1**…………………………...8

Figure S7. ^{1}H-^{1}H COSY (DMSO-d_6) spectrum of compound **1**……………………..9

Figure S8. ROESY (DMSO-d_6) spectrum of compound **1**……………………….....10

Figure S9. UV spectrum of compound **1**…………………………………………....11

Figure S10. IR spectrum of compound **1**…………………………………………....12

Figure S11. HRESI-MS spectrum of compound **20**……………………………….....13

Figure S12. ^{1}H-NMR (CD_3OD) spectrum of compound **20**………………..………14

Figure S13. ^{13}C-NMR (CD_3OD) spectrum of compound **20**………………………...15

Figure S14. DEPT (90°,135°) spectrum of compound **20**…………………...…....16

Figure S15. HSQC (CD_3OD) spectrum of compound **20**…………………………....17

Figure S16. HMBC (CD_3OD) spectrum of compound **20**……………...………...18

Figure S17. ^{1}H-^{1}H COSY (CD_3OD) spectrum of compound **20**……….…………...19

Figure S18. UV spectrum of compound **20**…………………………...………..20

Figure S19. IR spectrum of compound **20**……………………………………….21

Figure S20. The effects of compounds on DNA cleavage…………………………22

Figure S21 Results of flavonoid **5** induced cell cycle arrests in HCT116 cells…......23

Spectra data of compounds 2-19 and 21-29…………………………………24

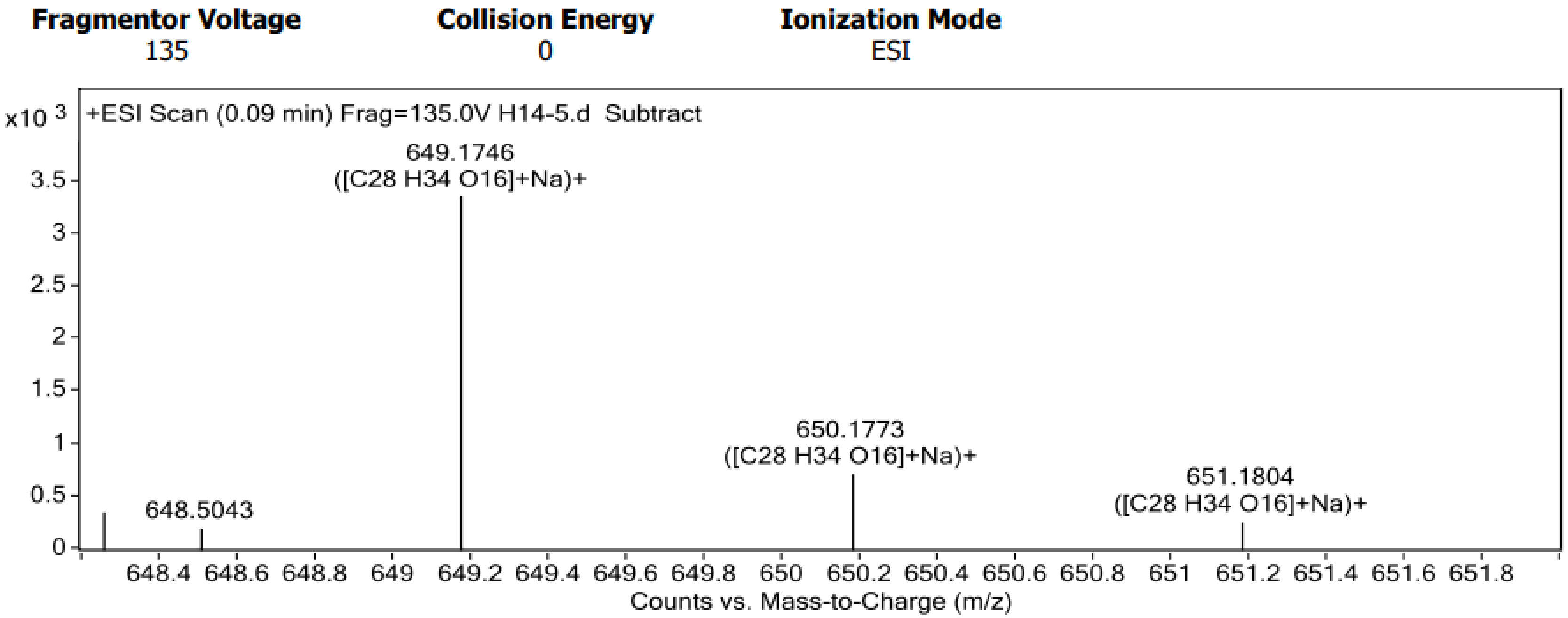

Figure S1. HRESI-MS spectrum of compound **1**

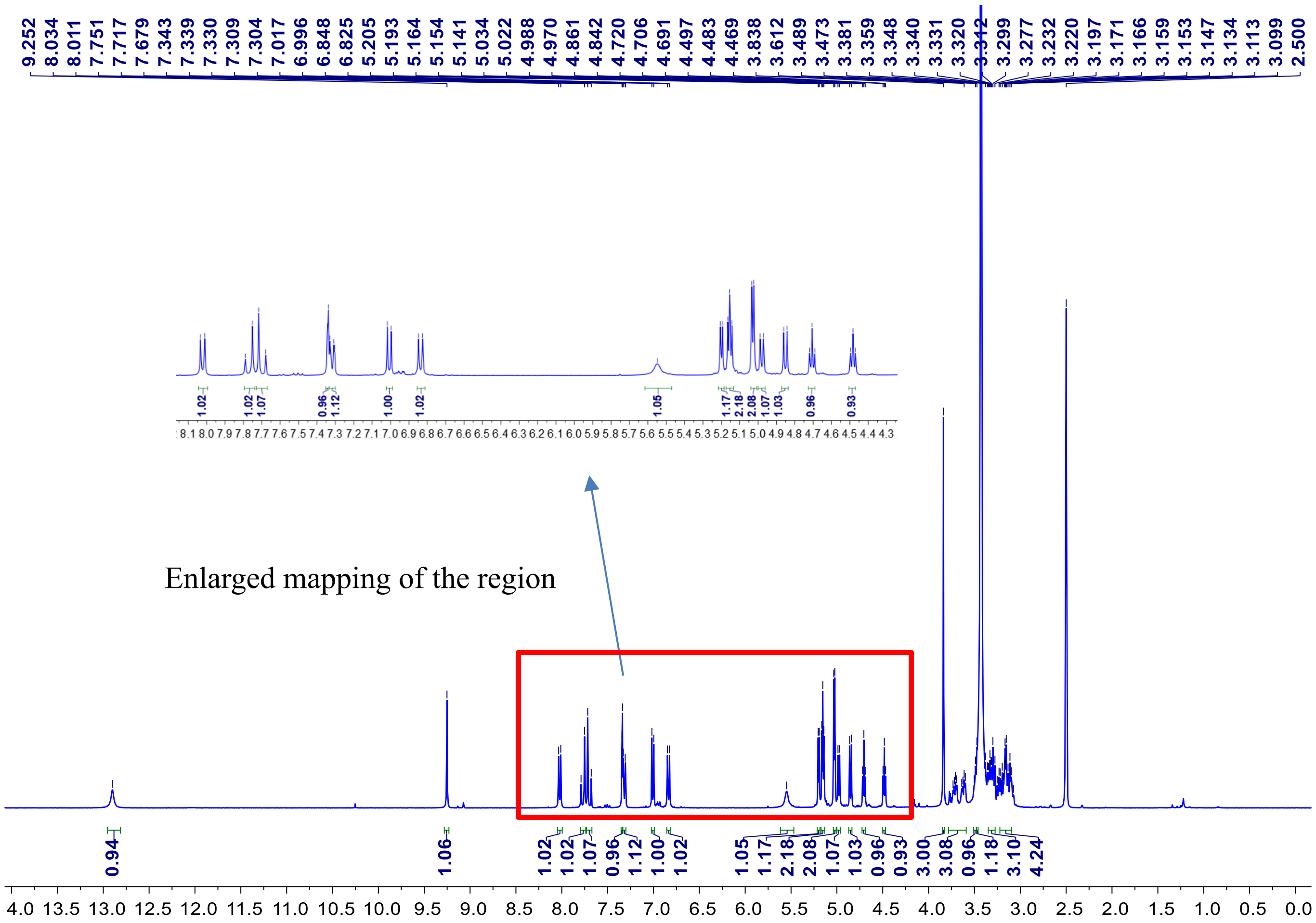

Figure S2. ^{1}H-NMR (DMSO-*d*6) spectrum of compound **1**

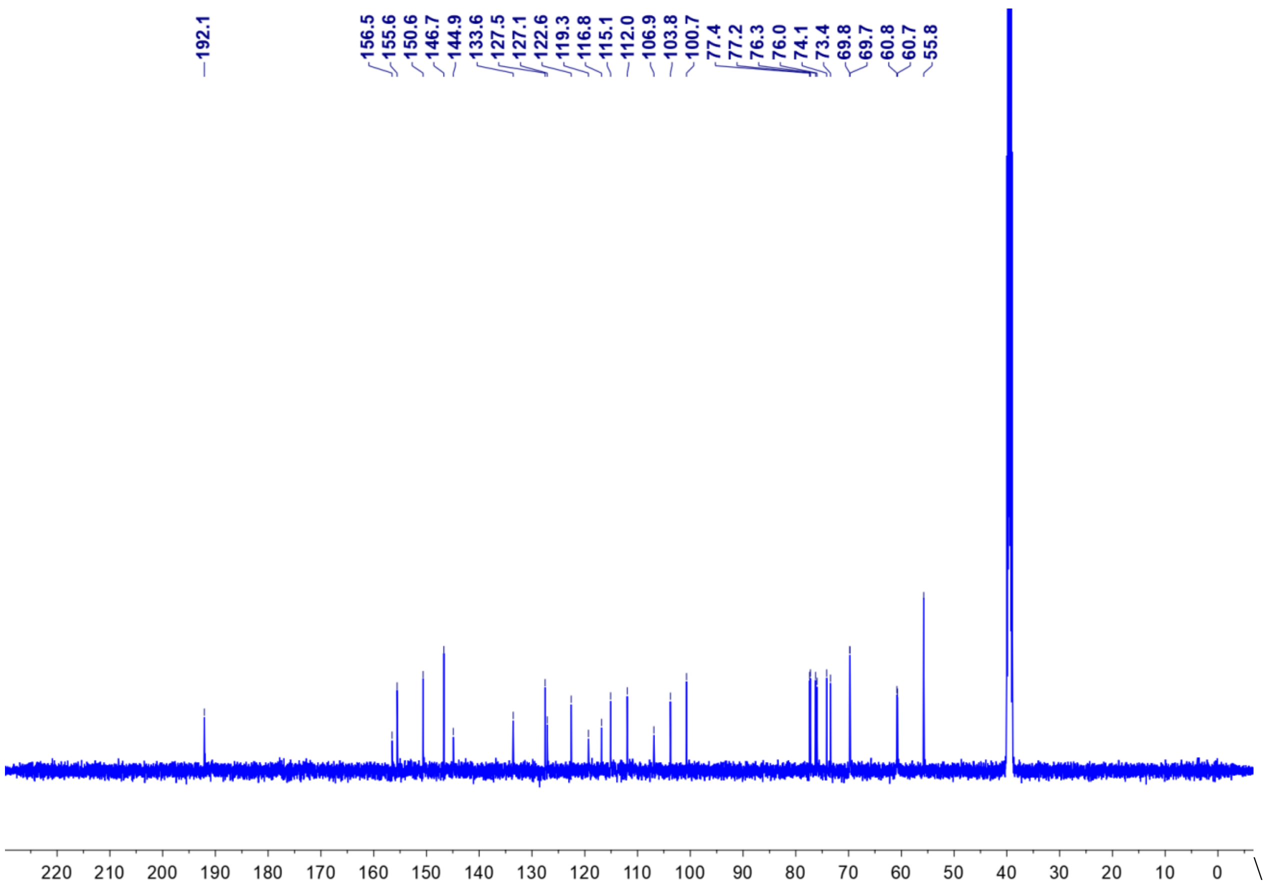

Figure S3. ^{13}C-NMR (DMSO-*d*6) spectrum of compound **1**

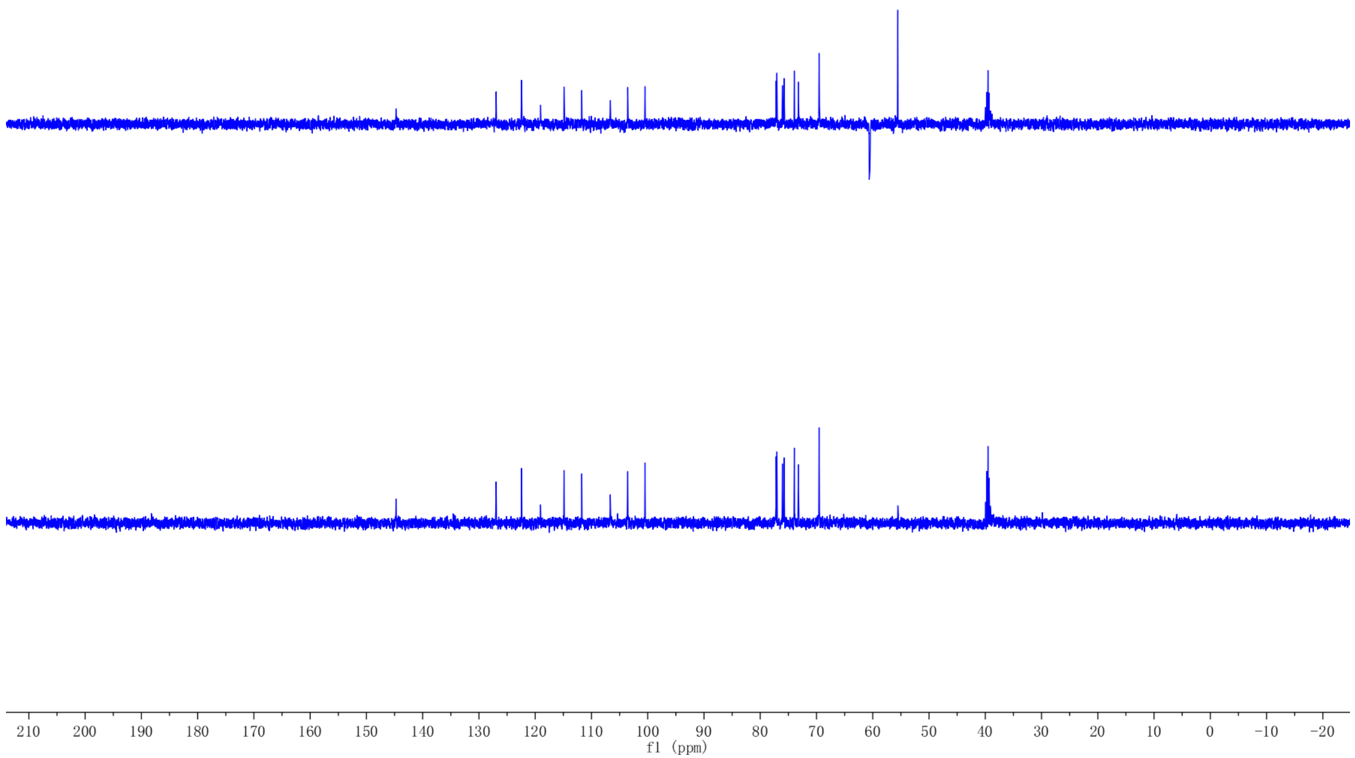

Figure S4. DEPT (90°,135°) spectrum of compound **1**

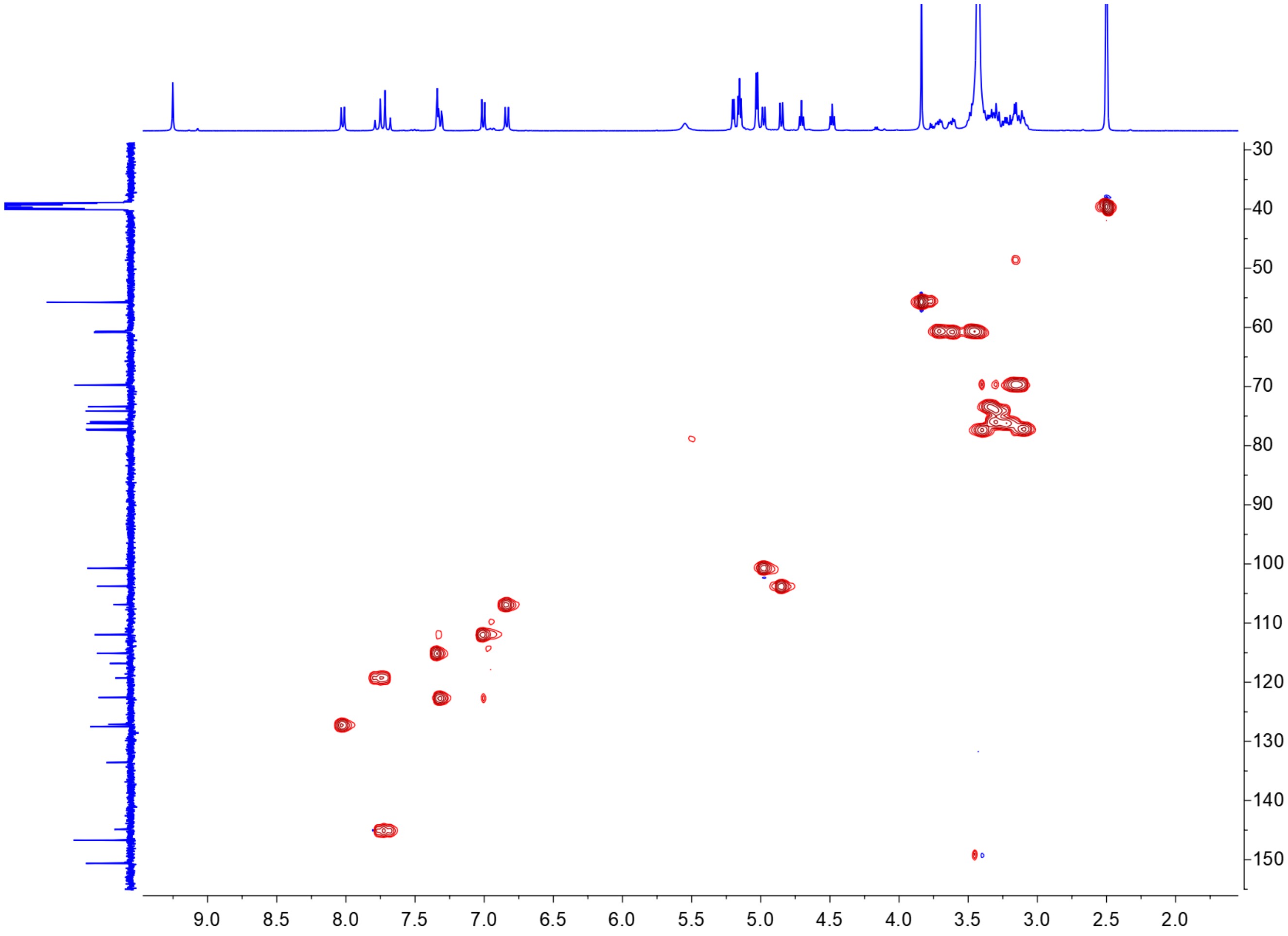

Figure S5. HSQC (DMSO-d6) spectrum of compound **1**

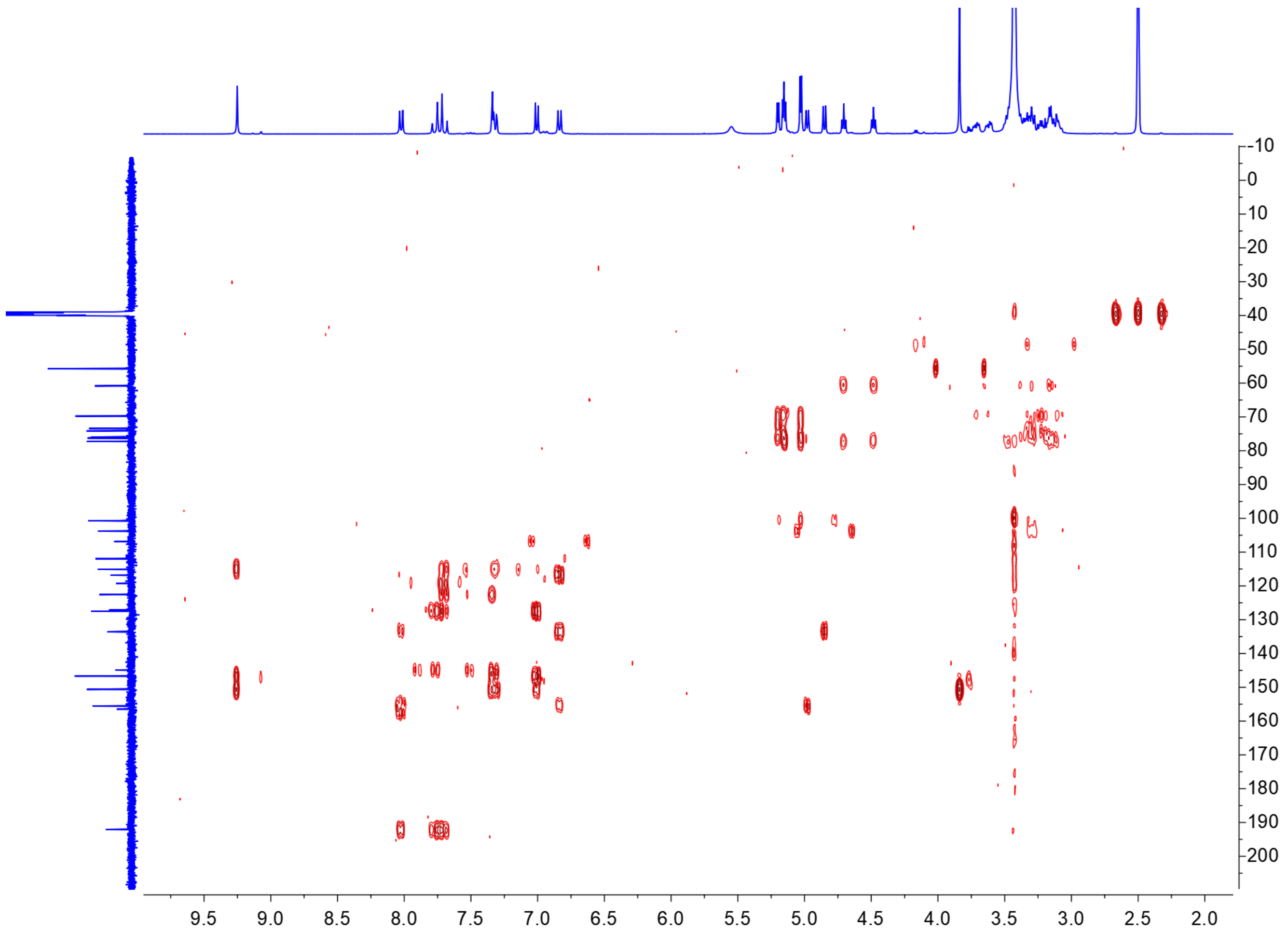

Figure S6. HMBC (DMSO-*d6*) spectrum of compound **1**

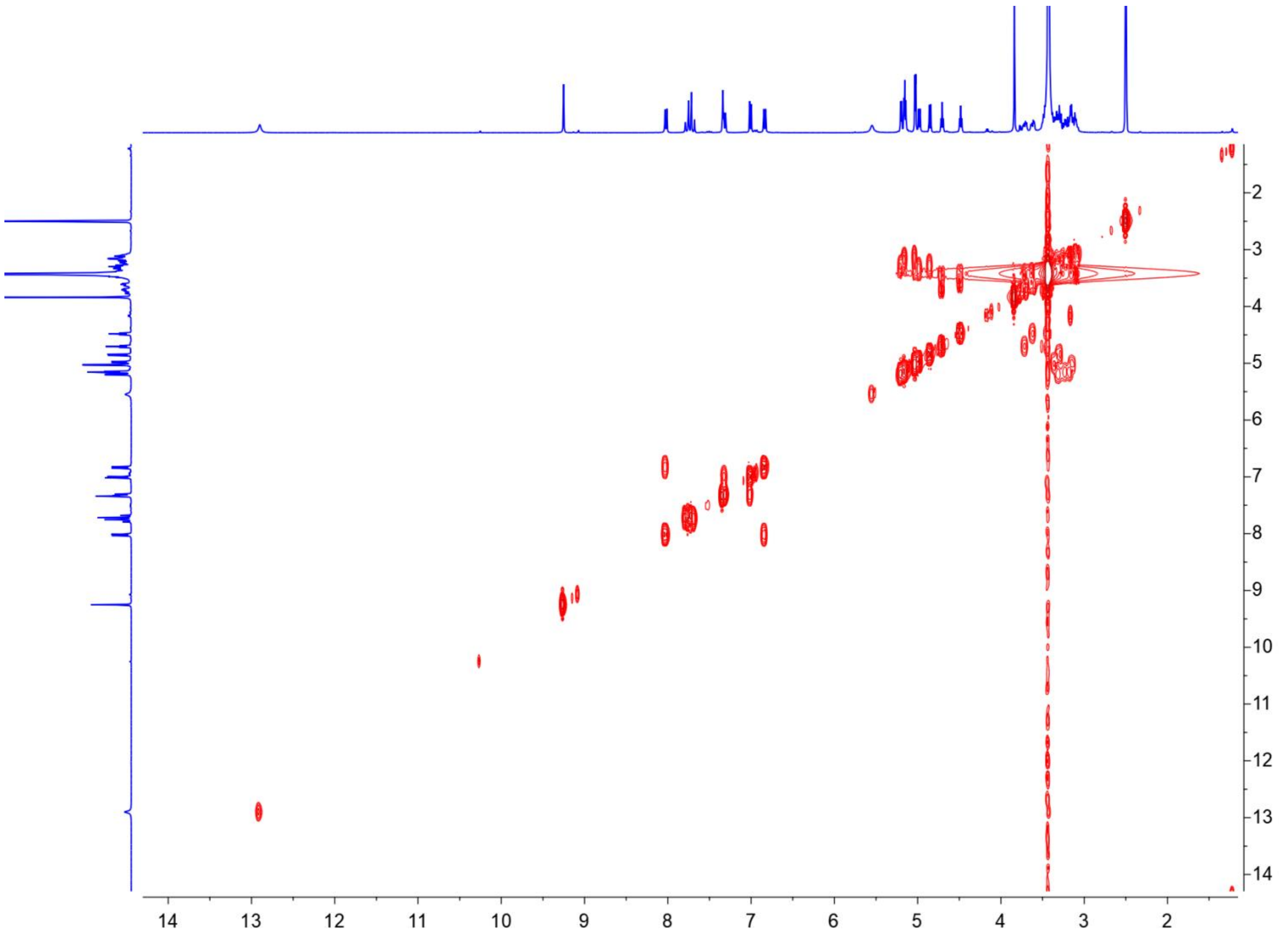

Figure S7. ^{1}H-^{1}H COSY (DMSO-*d6*) spectrum of compound **1**

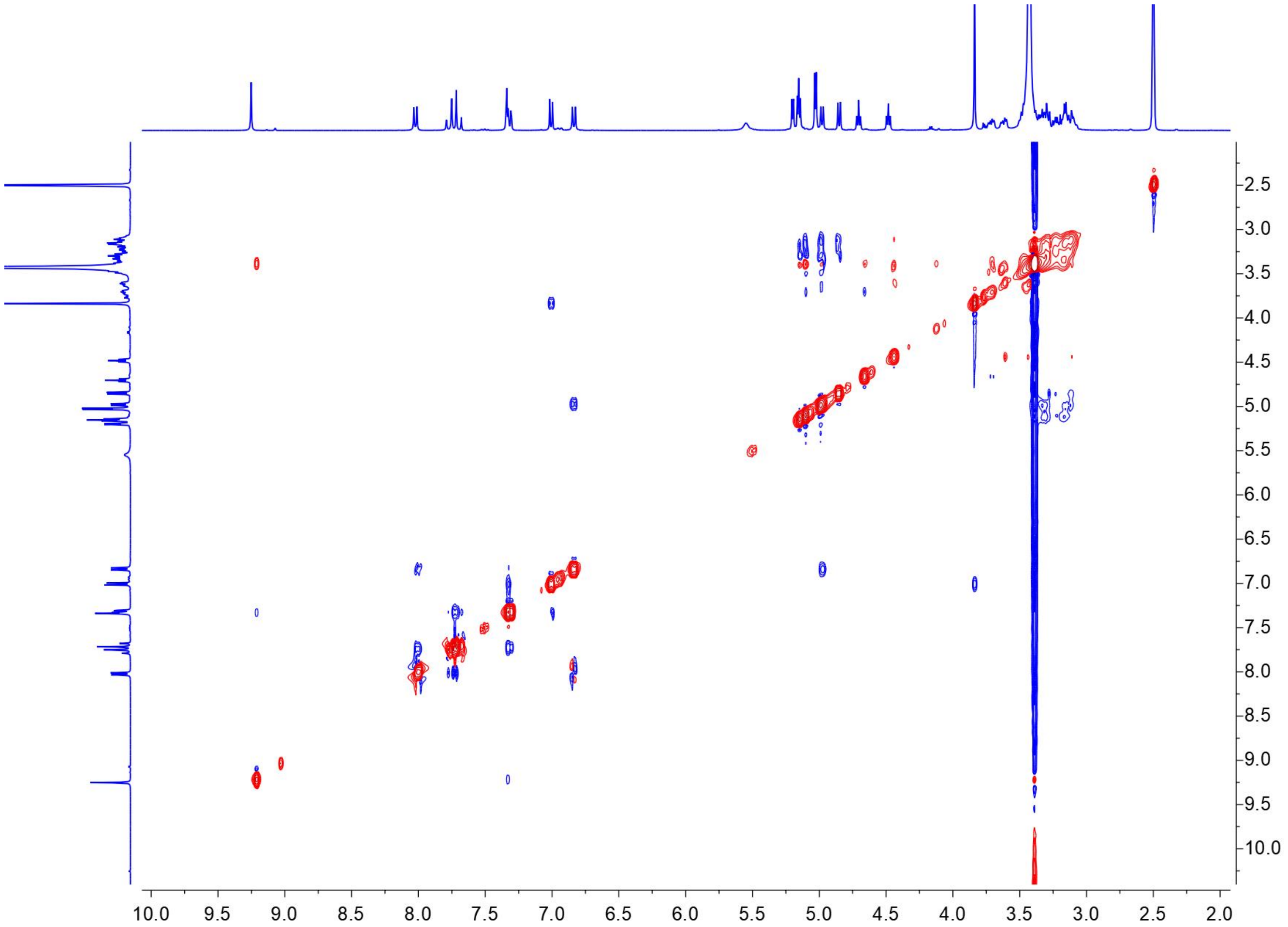

Figure S8. ROESY (DMSO-*d6*) spectrum of compound **1**

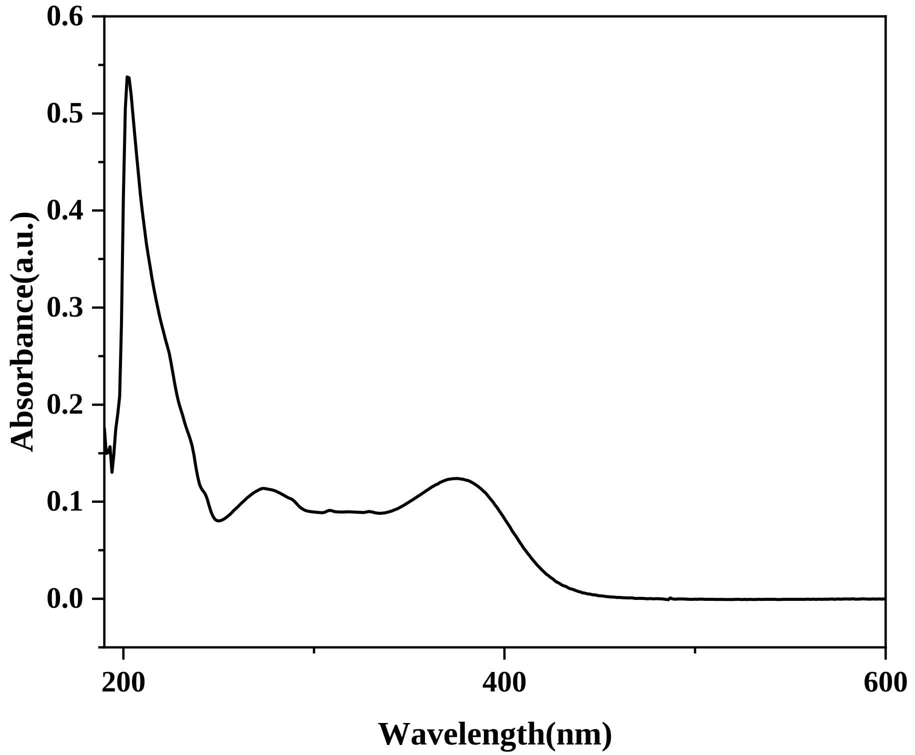

Figure S9. UV splectrum of compound **1**

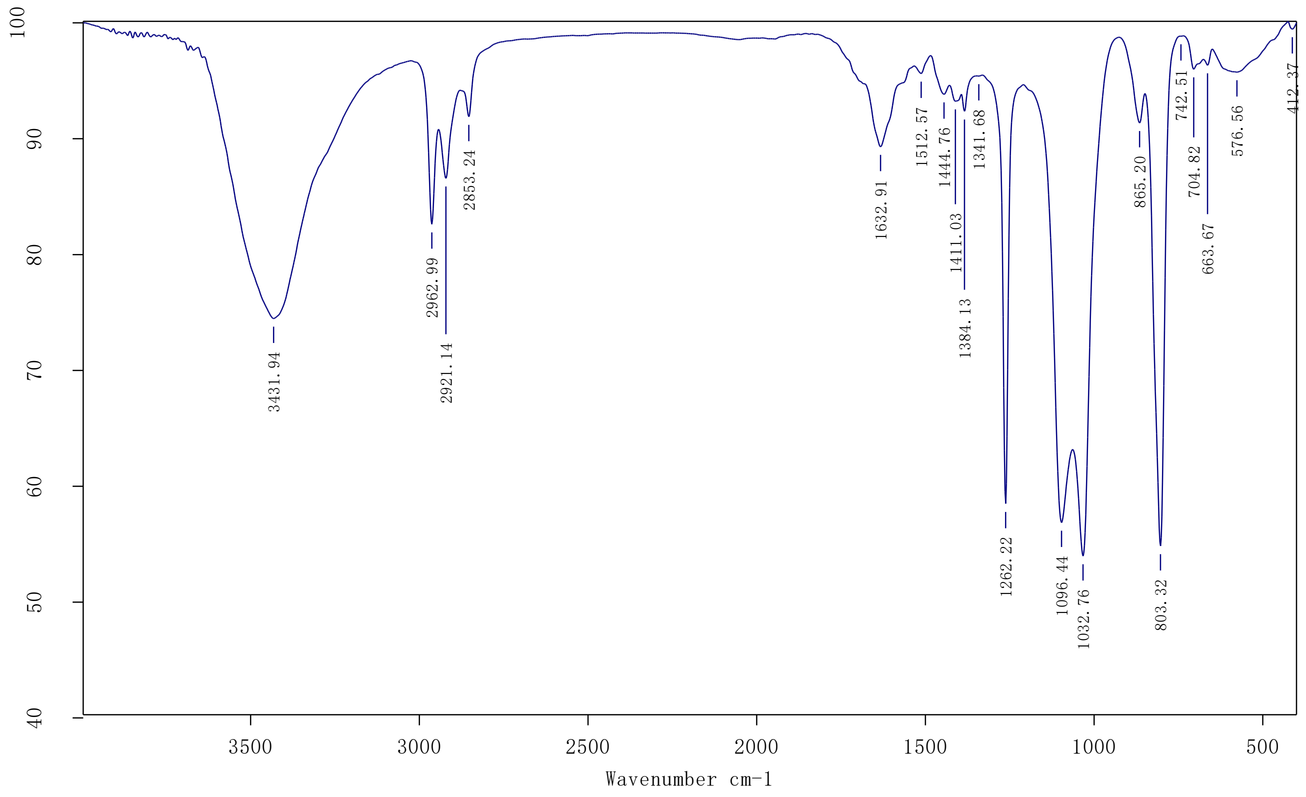

Sample Name: 14-5
Sample Form: KBr
Path of File: E:\data
Date of Measurement: 2024/5/22

Resolution: 4
Aperture Setting: 6 mm
Number of Background Scans: 16
Number of Sample Scans: 16

Beamsplitter Setting: KBr
Source Setting: MIR
Instrument Type: BRUKER VERTEX 70
Soft Version: OPUS8.1

Figure S10. IR splectrum of compound **1**

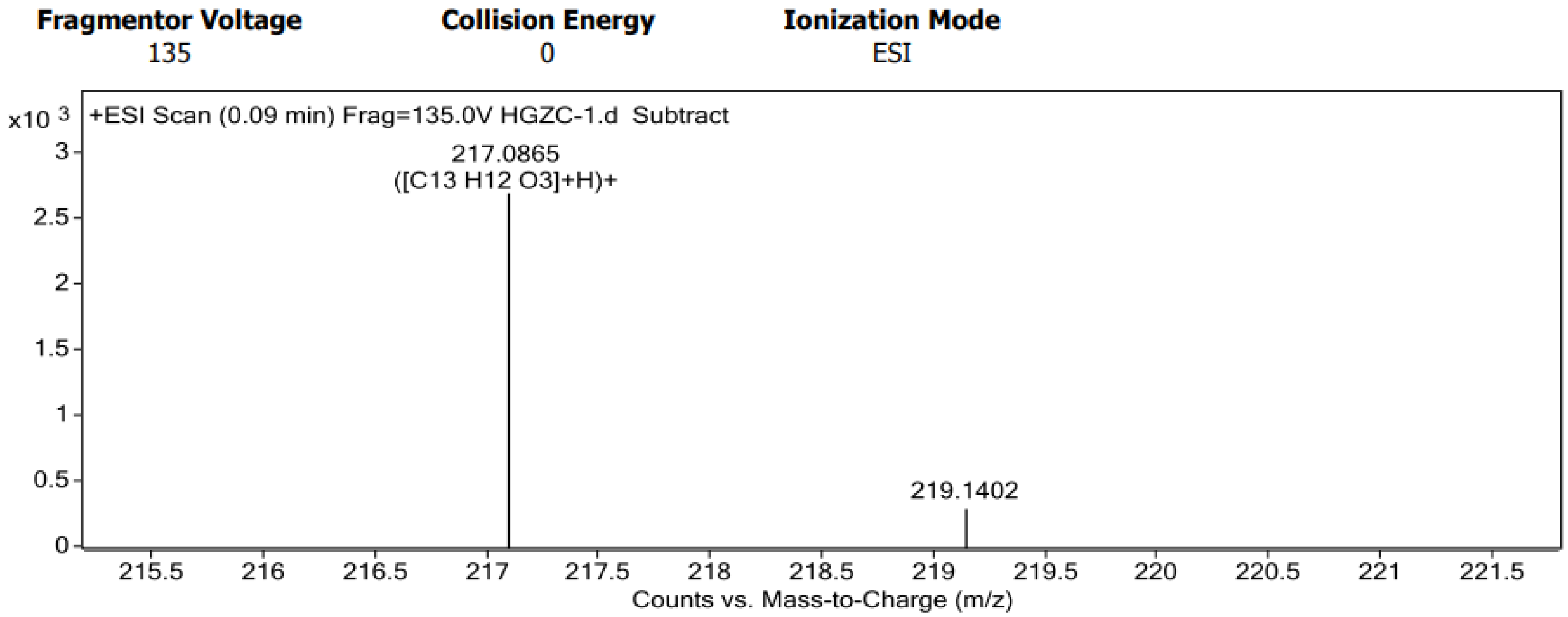

Figure S11. HRESI-MS spectrum of compound **20**

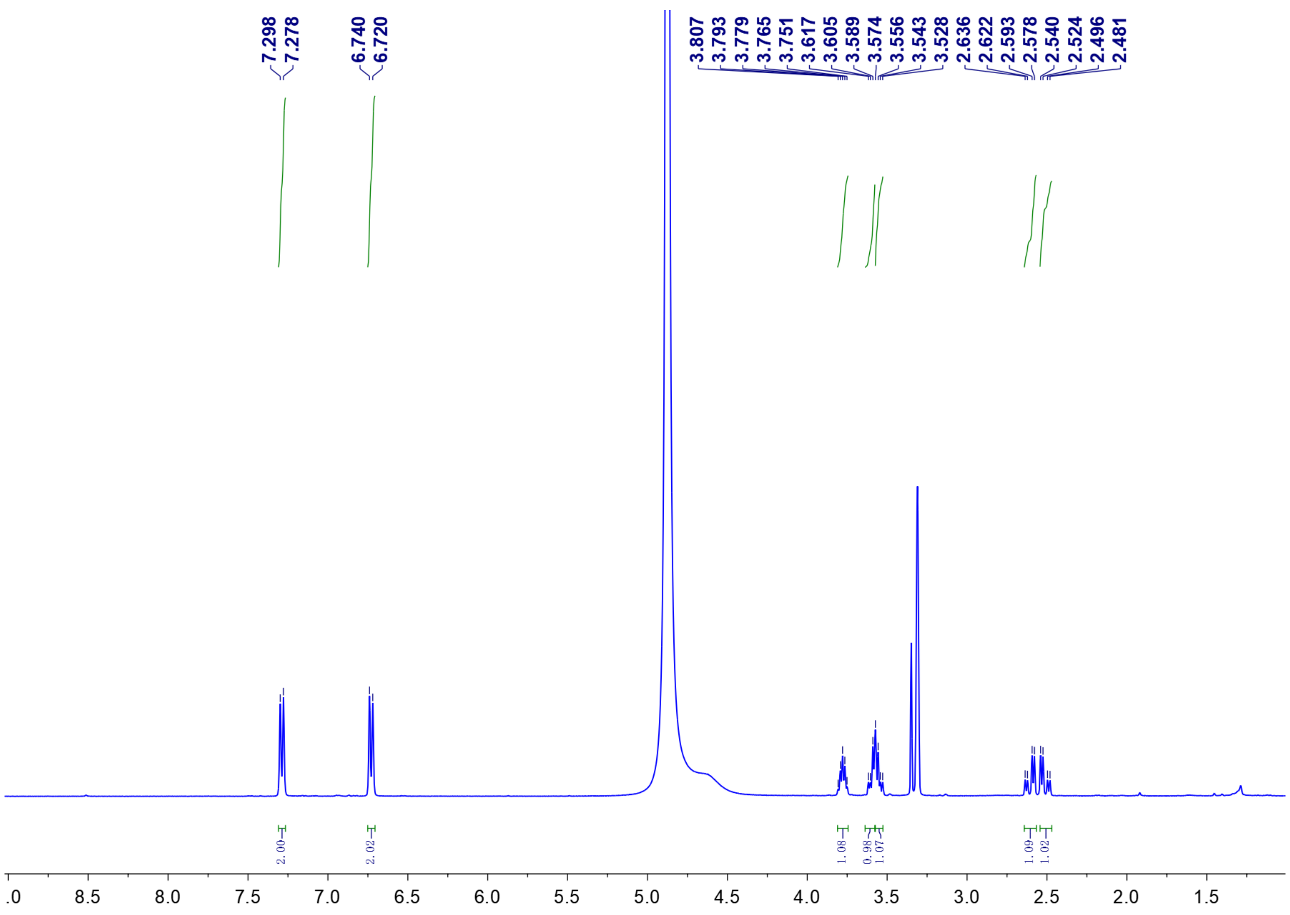

Figure S12. ^{1}H-NMR (CD_3OD) spectrum of compound **20**

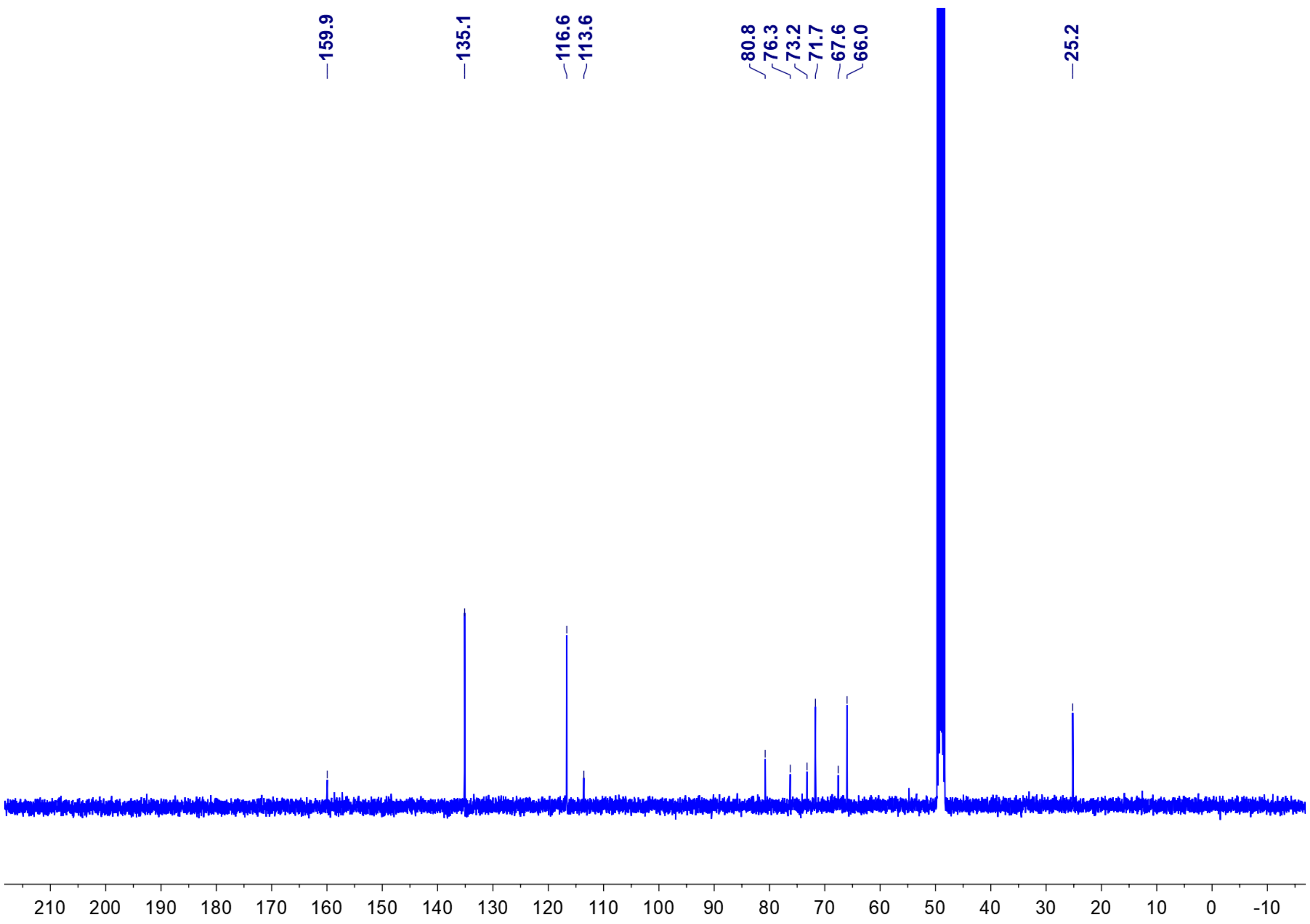

Figure S13. ^{13}C-NMR (CD_3OD) spectrum of compound **20**

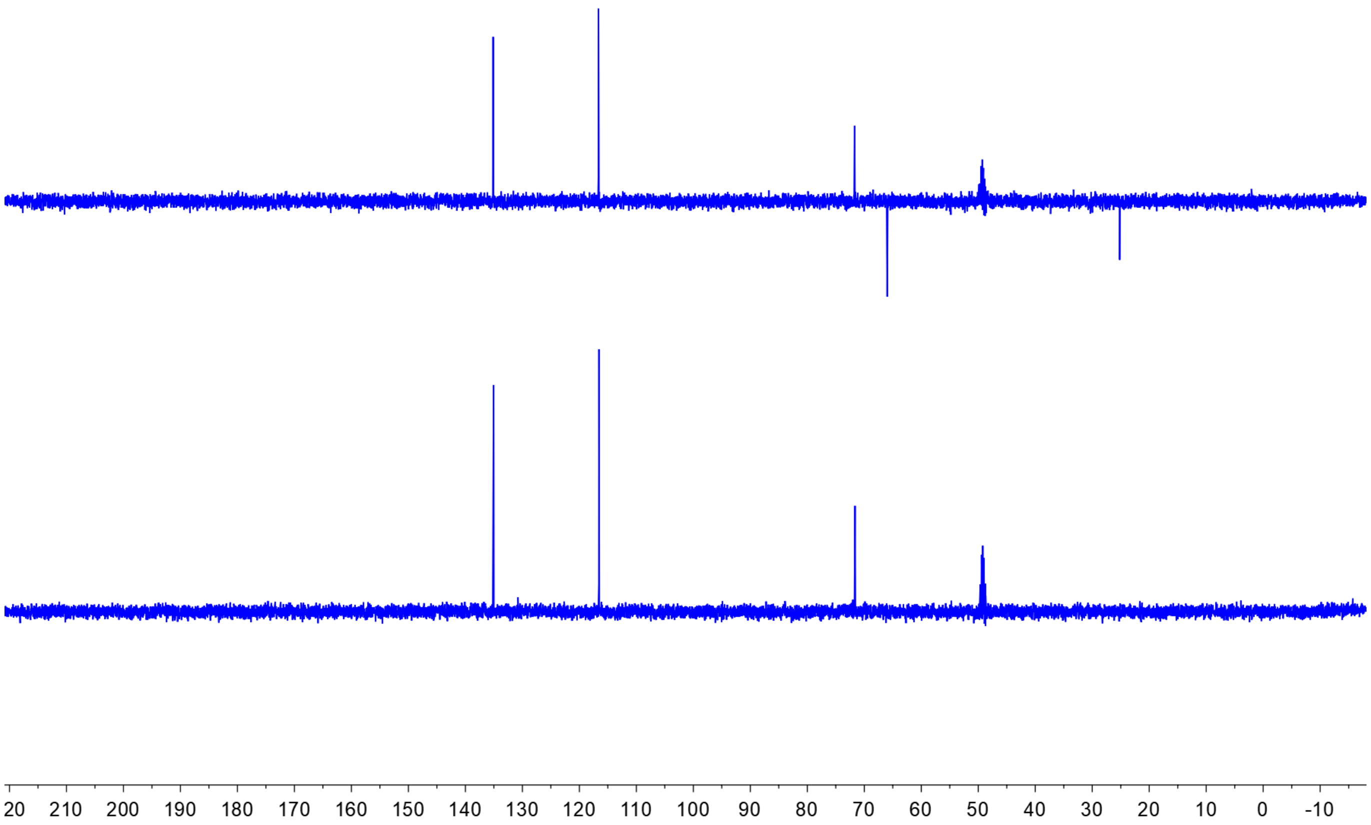

Figure S14. DEPT (90’,135’) spectrum of compound **20**

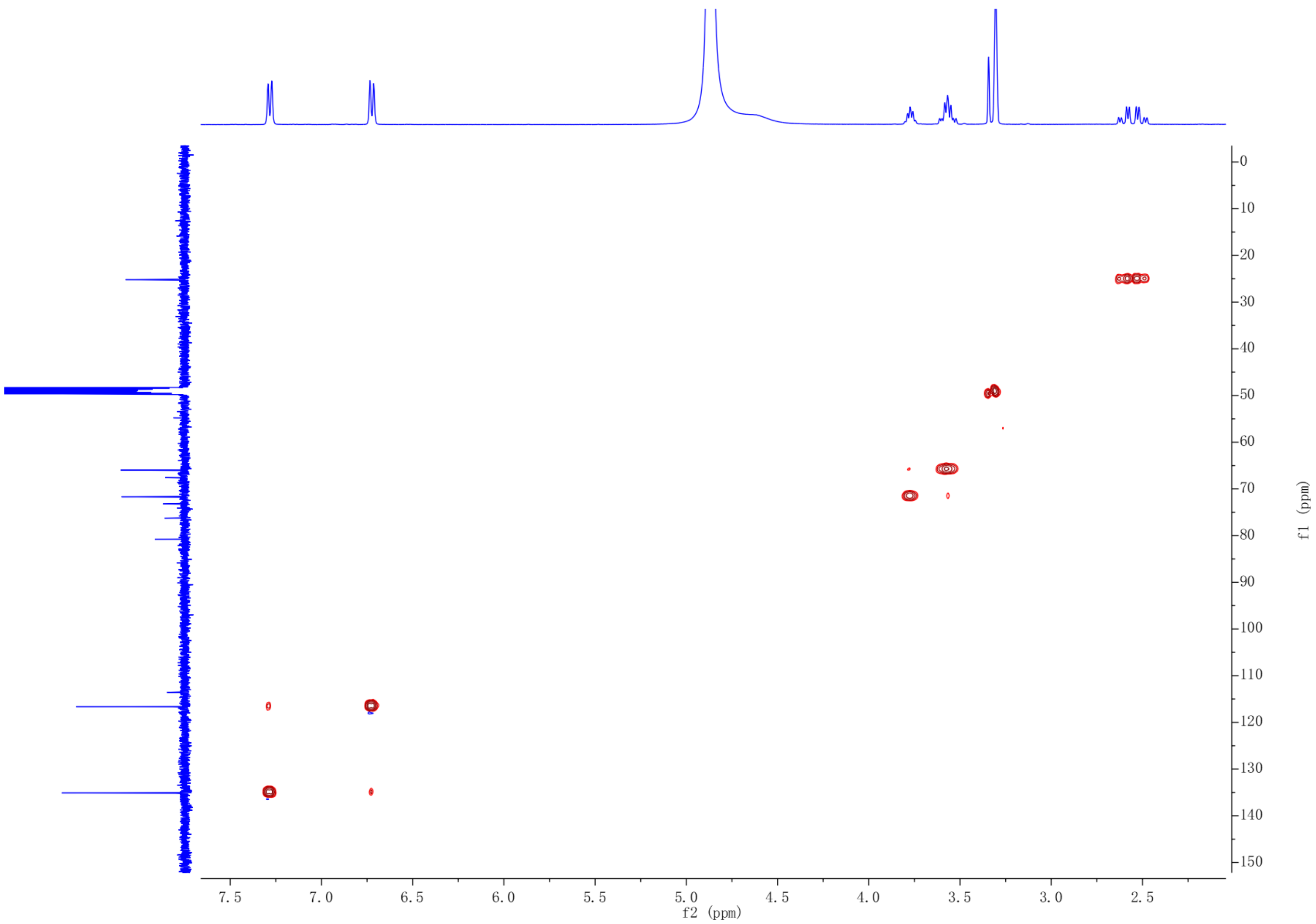

Figure S15. HSQC (CD_3OD) spectrum of compound **20**

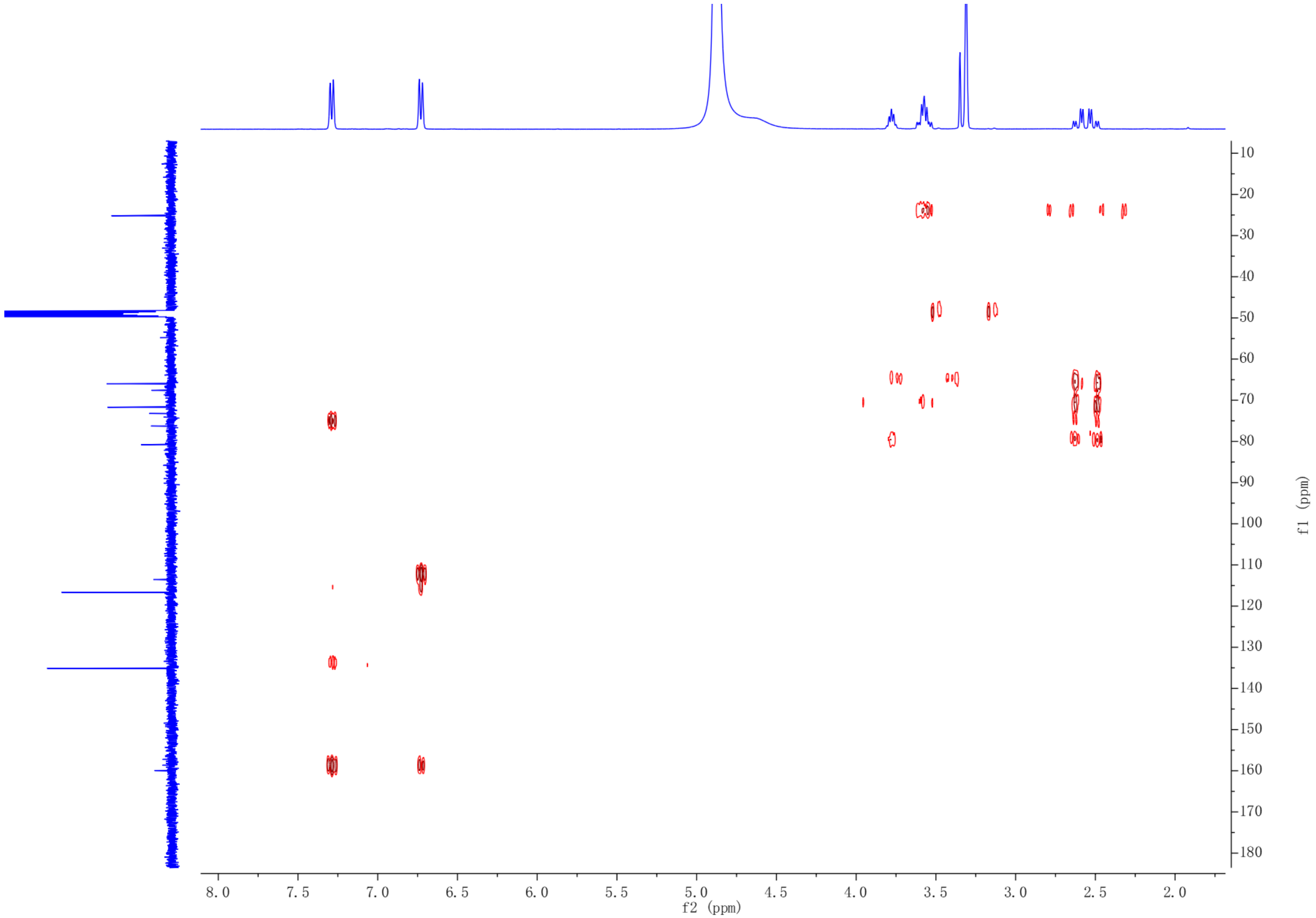

Figure S16. HMBC (CD_3OD) spectrum of compound **20**

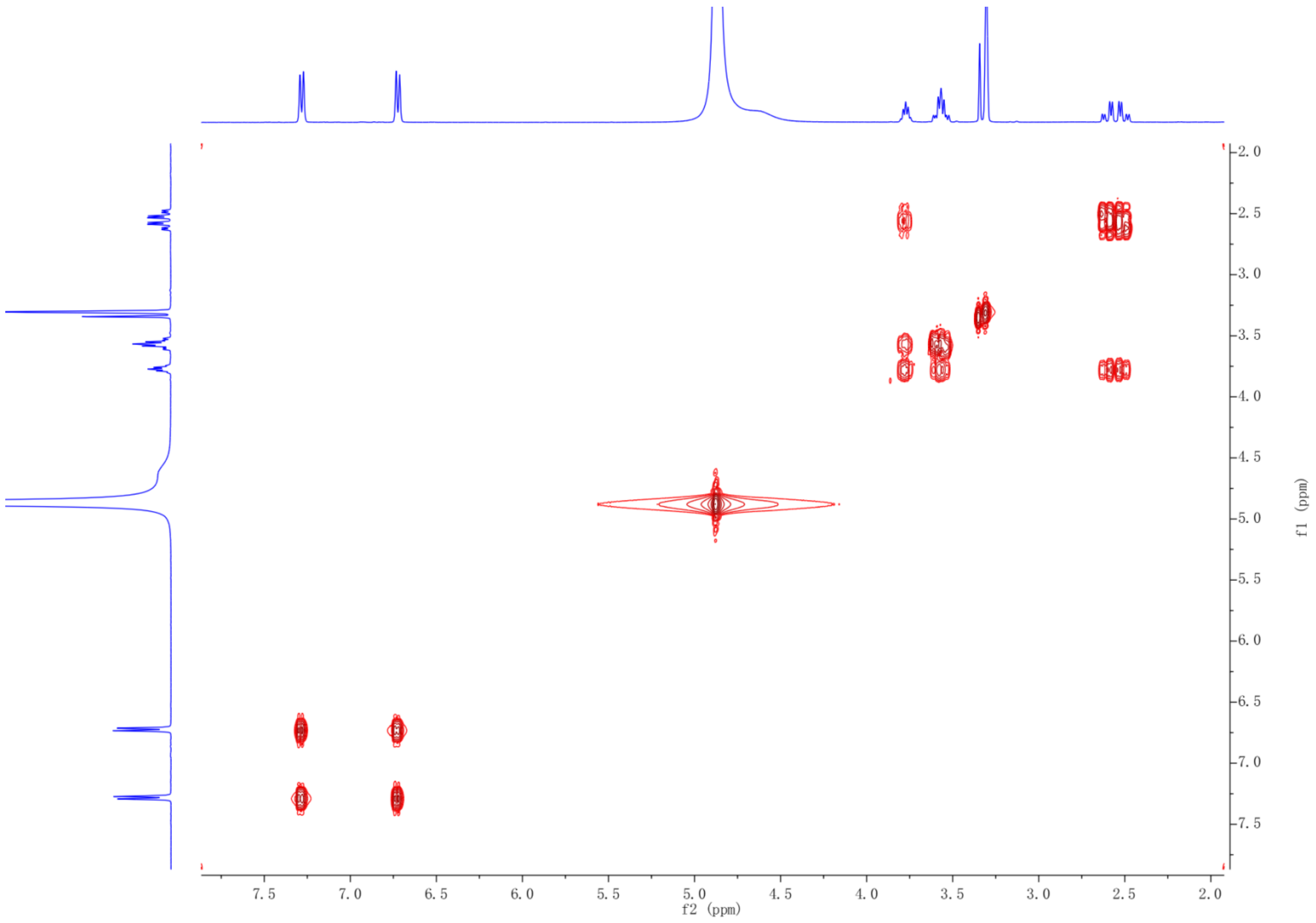

Figure S17. ^{1}H-^{1}H COSY (CD3OD) spectrum of compound **20**

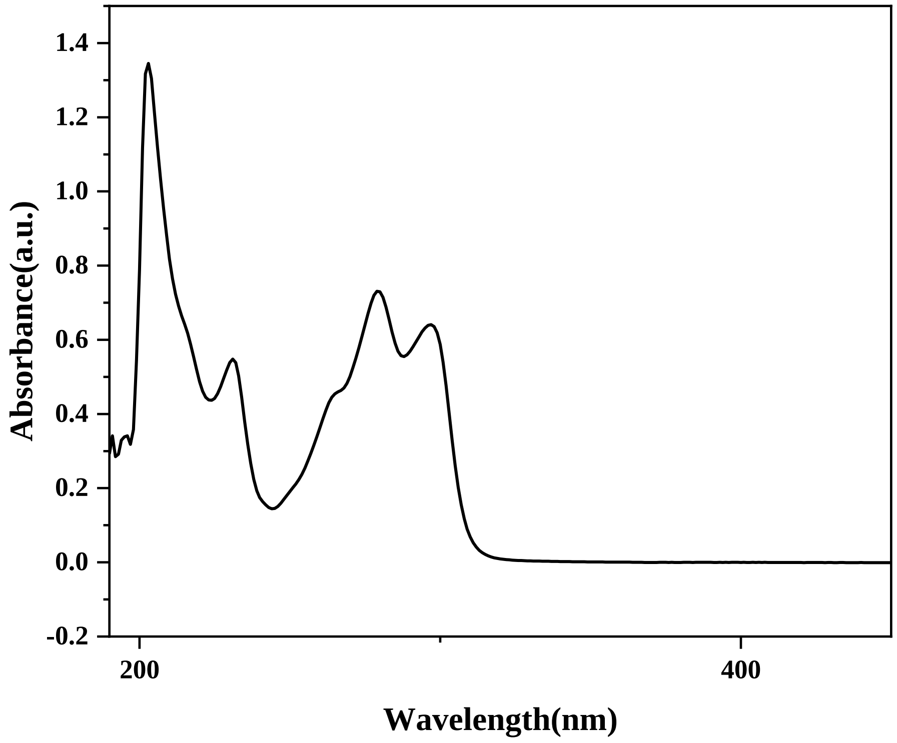

Figure S18. UV spectrum of compound **20**

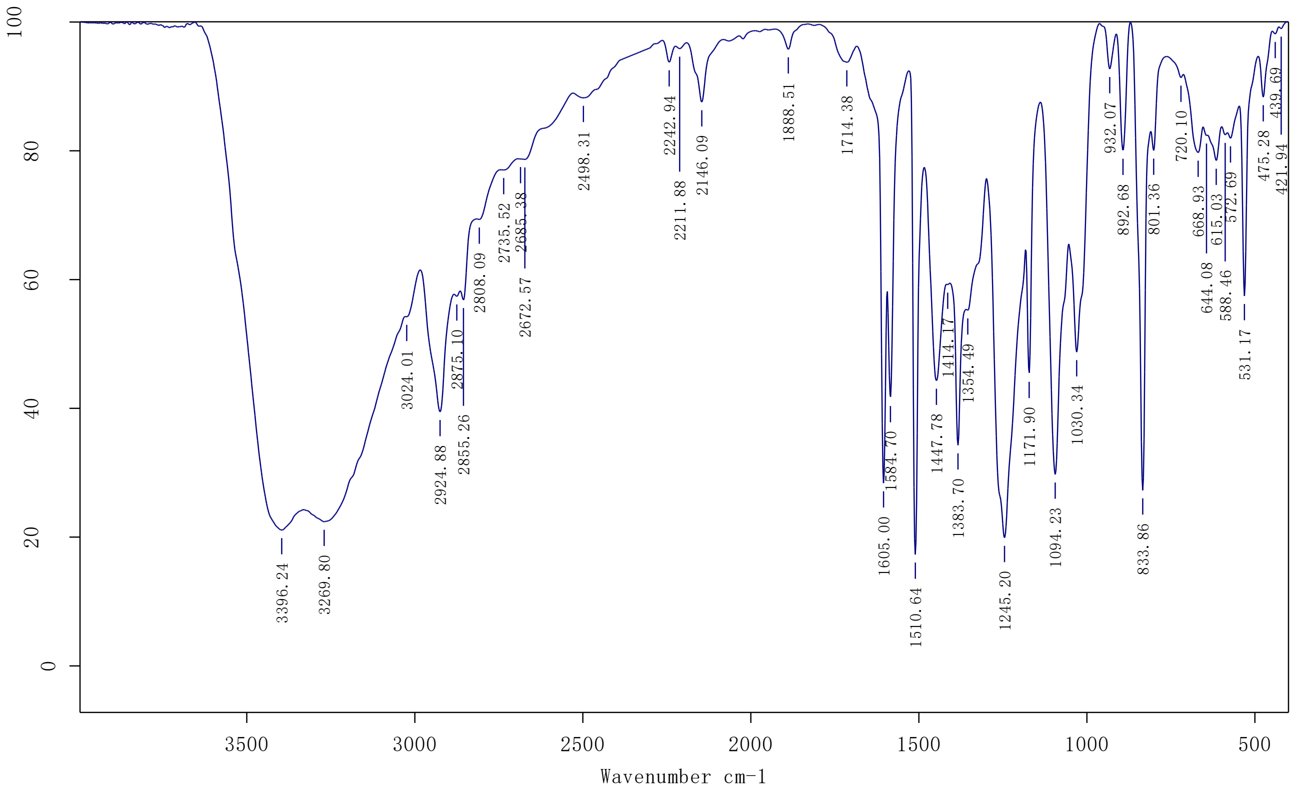

Sample Name: G2C-1
Sample Form: KBr
Path of File: E:\data
Date of Measurement: 2024/5/22

Resolution: 4
Aperture Setting: 6 mm
Number of Background Scans: 16
Number of Sample Scans: 16

Beamsplitter Setting: KBr
Source Setting: MIR
Instrument Type: BRUKER VERTEX 70
Soft Version: OPUS8.1

Figure S19. IR spectrum of compound **20**

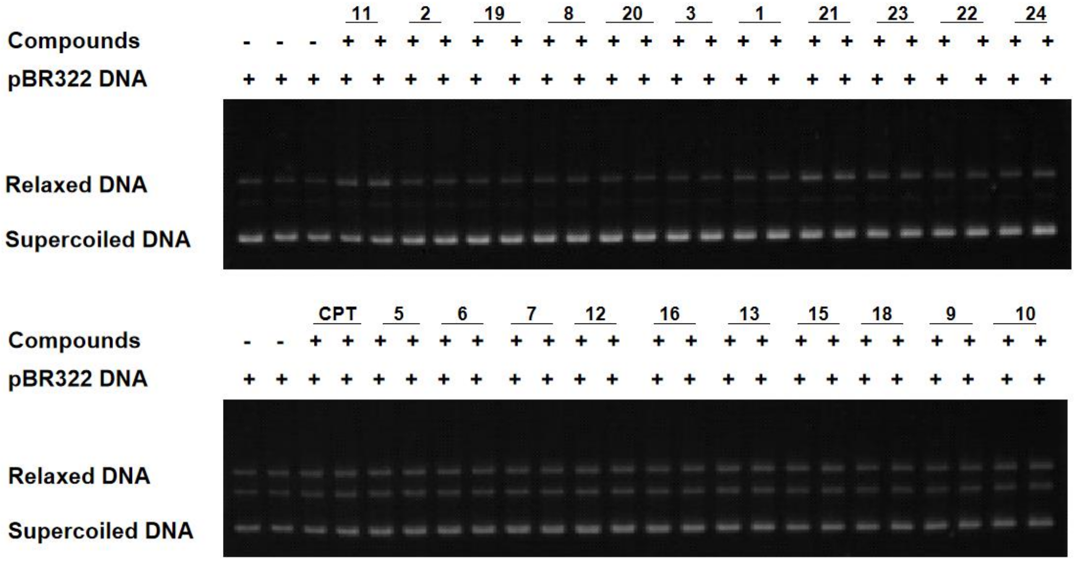

Figure S20. The effects of compounds on DNA cleavage. The tested concentration of the compounds is 400 μM.

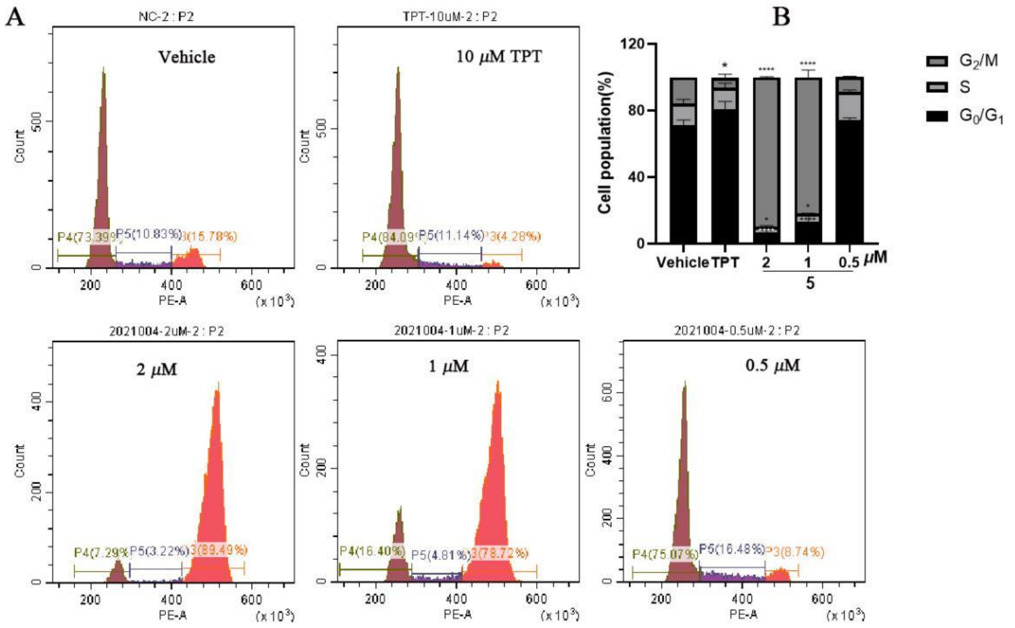

Figure S21 Results of flavonoid **5** induced cell cycle arrests in HCT116 cells. A) Representative images of flow-cytometry analysis of DNA content; B) Statistic analysis of cells in G_1, S, and G_2/M phase after treated with **5**. After treatment with 2 and 1 μM compounds **5** for 24 h, the percentages of cell population in the G_2/M phase significantly increased from 15.77% to 89.72% and 81.84%, and the cells in G_1/S (combined) phase remarkably decreased from 84.08% to 10.16% and 18.13%, respectively, which meant a potent G_2/M cell cycle arrest caused by the treatment ($P<$ 0.0001). In addition, after treatment with 0.5 μM compounds **5** for 24 h, the percentages of cells in the G_1/S phase increased from 84.08% to 91.23% ($P<$ 0.01), which indicated that an apparent G_1 or S phase arrest was induced after the treatment with a lower compound concentration. *p < 0.05, ***p < 0.001, and ****p < 0.0001 significantly different from the vehicle control. Data are expressed as mean ± SD values from three independent experiments.

Spectra data of compounds 2-19 and 21-29

Okanin-4-methyl ether-3'-*O*-*β*-*D*-glucopyranoside (**2**): Yellow amorphous powder. ^{1}H-NMR (400 MHz, CD_4O) δ (ppm): 7.79 (1H, d, J = 8.8 Hz, H-6'), 7.72 (1H, d, J = 15.6 Hz, H-β), 7.54 (1H, d, J = 15.2 Hz, H-α), 7.20 (1H, d, J = 1.8 Hz, H-2), 7.17 (1H, dd, J = 8.4, 1.8 Hz, H-6), 6.93 (1H, d, J = 8.4 Hz, H-5), 6.48 (1H, d, J = 9.2 Hz, H-5'), 4.80 (1H, d, J = 7.6 Hz, H-1"), 3.87 (3H, s, -OMe), 3.78 (1H, dd, J = 12.0, 2.4 Hz, H-6a"), 3.71 (1H, dd, J = 12.0, 5.6 Hz, H-6b"), 3.52 (1H, m, H-5″), 3.42～3.49 (2H, m, 2″,3″), 3.27 (1H, m, 4″); ^{13}C-NMR (100 MHz, CD_4O) δ (ppm): 193.7 (C=O), 159.2 (C-4′), 158.9 (C-2′), 151.9 (C-4), 148.0 (C-3), 146.1 (C-β), 133.7 (C-3′), 129.4 (C-1), 128.9 (C-6′), 123.7 (C-6), 119.1 (C-α), 115.4 (C-2), 115.3 (C-1′), 112.5 (C-5), 109.5 (C-5′), 106.6 (C-1″), 78.3 (C-3″), 77.6 (C-5″), 75.3 (C-2″), 70.7 (C-4″), 61.9 (C-6″), 56.4 (4-OMe). Compound **2** was identified as okanin-4-methyl ether-3'-*O*-*β*-*D*-glucopyranoside, comparing the physical and spectral data with the reported data [17].

2'-hydroxy-4,4'-dimethoxy-chalcone (**3**): 2'-hydroxy-4,4'-dimethoxy-chalcone (**3**): Yellow amorphous powder. ^{1}H-NMR (400 MHz, $CDCl_3$) δ (ppm): 13.57 (1H, s, OH), 7.83 (1H, d, J = 15.6 Hz, H-1β), 7.78 (1H, d, J = 8.4 Hz, H-6'), 7.56 (2H, d, J = 8.2 Hz, H-2, H-6), 7.42 (1H, d, J = 15.6 Hz, H-1α), 6.91 (2H, d, J = 8.3 Hz, H-3, H-5), 6.49 (1H, d, J = 8.3 Hz, H-5'), 6.43 (1H, s, H-3'), 3.85 (6H, d, J = 1.9 Hz, H-4, H-4'). ^{13}C-NMR (100 MHz, $CDCl_3$) δ (ppm): 191.9 (C-1β'), 166.6 (C-4'), 166.1 (C-6'), 161.8 (C-4), 144.3 (C-1β), 131.1 (C-2'), 130.4 (C-2, C-6), 127.5 (C-1), 117.8 (C-1α), 114.5 (C-3, C-5), 114.2 (C-1'), 107.6 (C-3'), 101.1 (C-5'), 55.6 (C-4), 55.4 (C-4'). Compound **3** was identified as 2'-hydroxy-4, 4'-dimethoxy-chalcone, comparing the physical and spectral data with the reported data [18].

3,7-dimethylquercetin (**4**): Yellow amorphous powder. ^{1}H-NMR (400 MHz, CD_4O) δ (ppm): 7.67 (1H, d, J = 2.2 Hz, H-2'), 7.58 (1H, dd, J = 8.5, 2.2 Hz, H-6'), 6.95 (1H, d, J = 8.5 Hz, H-5'), 6.58 (1H, s, J = 2.2 Hz, H-8), 6.35 (1H, d, J = 2.2 Hz, H-6), 3.89 (3H, s, 3-OCH_3), 3.84 (3H, s, 7-OCH_3). ^{13}C-NMR (100 MHz, CD_4O) δ (ppm): 180.3 (C-4), 166.0 (C-7), 163.1 (C-5), 161.7 (C-10), 158.7 (C-9), 151.7 (C-2), 150.3 (C-4'), 147.6 (C-3'), 139.1 (C-3), 122.6 (C-6'), 122.1 (C-1'), 116.6 (C-5'), 116.1 (C-2'), 99.8 (C-6), 95.0 (C-8), 61.0 (3-OCH_3), 56.4 (7-OCH_3). Compound **4** was identified as 3,7-dimethylquercetin, comparing the physical and spectral data with the reported data [19].

Centaureidin (**5**): Yellow amorphous powder. ^{1}H-NMR (400 MHz, CD_4O) δ (ppm): 7.72 (1H, dd, J = 8.6, 2.2 Hz, H-6'), 7.69 (1H, d, J = 2.2 Hz, H-2'), 7.32 (1H, d, J = 8.6 Hz, H-5'), 3.97 (3H, s, 4'-OCH_3), 6.56 (1H, s, H-8), 3.95 (3H, s, 6-OCH_3), 3.87 (3H, s, 3-OCH_3). ^{13}C-NMR (100MHz, CD_4O) δ (ppm): 180.2 (C-4), 158.8 (C-7), 157.5 (C-2), 153.7 (C-9), 151.6 (C-5、C-4'), 147.5 (C-3'), 139.5 (C-3), 132.5 (C-6), 124.1 (C-1'), 122.1 (C-6'), 116.1 (C-2'), 112.2 (C-5'), 106.3 (C-10), 95.0 (C-8), 61.0 (6-OCH_3), 60.6 (3-OCH_3), 56.4 (4'-OCH_3). Compound **5** was identified as centaureidin, comparing the physical and spectral data with the reported data [20].

6,8,3'-trihydroxy-3,7,4'-trimethoxy-flavone (**6**): Yellow amorphous powder. ^{1}H-NMR (400 MHz, CD_4O) δ (ppm): 7.59 (1H, dd, J = 8.0 Hz, 2.0 Hz, H-6'), 7.57 (1H, d, J = 2.0 Hz, H-2'), 7.04 (1H, d, J = 8.2 Hz, H-5'), 6.87 (1H, s, H-5), 3.95 (3H, s, 4'-OCH_3), 3.89 (3H, s, 7-OCH_3), 3.83 (3H, s, 3-OCH_3). ^{13}C-NMR (100 MHz, CD_4O) δ (ppm): 180.3 (C-4), 158.9 (C-2), 157.7 (C-6), 153.8 (C-8), 151.7 (C-9), 147.6 (C-4'), 139.5 (C-3'), 139.3 (C-3), 132.6 (C-7), 124.2 (C-1'), 122.2 (C-6'), 116.1 (C-2'), 112.3 (C-4'), 106.4 (C-10), 95.0 (C-5), 61.0 (7-OCH_3), 60.6 (4'-OCH_3), 56.4 (3-OCH_3). Compound **6** was identified as 6,8,3'-trihydroxy-3,7,4'-trimethoxy-flavone, comparing the physical and spectral data with the reported data [21].

Jaceidin (**7**): Yellow amorphous powder. ^{1}H-NMR (400 MHz, CD_4O) δ (ppm): 7.75 (1H, d, J = 1.8 Hz, H-2'), 7.67 (1H, dd, J = 1.8, 8.4 Hz, H-6'), 6.97 (1H, d, J = 8.4 Hz, H-5'), 6.55 (1H, s, H-8), 3.97 (3H, s, 3'-OCH_3), 3.87 (3H, s, 6-OCH_3), 3.85 (3H, s, 3-OCH_3). ^{13}C-NMR (100MHz, CD_4O) δ (ppm): 180.3 (C-4), 159.0 (C-7), 157.7 (C-2), 153.8 (C-5), 151.7 (C-9), 148.9 (C-4'), 147.6 (C-3'), 139.3 (C-3), 132.6 (C-6), 124.2 (C-1'), 122.2 (C-6'), 116.2 (C-2'), 112.3 (C-5'), 106.4 (C-10), 95.1 (C-8), 61.0 (3-OCH_3), 60.6 (6-OCH_3), 56.4 (3'-OCH_3). Compound **7** was identified as jaceidin, comparing the physical and spectral data with the reported data [22].

Spinacetin (**8**): Yellow amorphous powder. ^{1}H-NMR (400 MHz, DMSO-d_6) δ (ppm): 12.35 (1H, s, 5-OH), 9.73 (1H, s, 7-OH), 9.33 (1H, s, 4'-OH), 8.66 (1H, s, 3-OH), 7.57 (1H, d, J = 2.0 Hz, H-2'), 7.46 (1H, dd, J = 8.0, 2.0 Hz, H-6'), 6.89 (1H, d, J = 8.0 Hz, H-5'), 6.80 (1H, s, H-8), 3.88 (3H, s, 6-OCH_3), 3.77 (3H, s, 3'-OCH_3); ^{13}C-NMR (100 MHz, DMSO-d_6) δ (ppm): 178.2 (C-4), 155.8 (C-7), 154.6 (C-9), 148.9 (C-2), 148.6 (C-5), 145.7 (C-3'), 145.3 (C-4'), 137.6 (C-3), 129.7 (C-6), 120.9 (C-6'), 120.6

(C-1'), 115.8 (C-2'), 115.5 (C-5'), 105.6 (C-10), 90.9 (C-8), 59.7 (3H, s, 6-OCH_3), 56.4 (3H, s, 3'-OCH_3). Compound **5** was identified as spinacetin, comparing the physical and spectral data with the reported data [23].

8,3'-dihydroxy-3,7,4'-trimethoxy-6-*O*-*β*-*D*-glucopyranosyl flavone (**9**): Yellow amorphous powder. ^{1}H-NMR (400 MHz, CD_4O) *δ* (ppm): 7.61 (1H, dd, *J* = 8.8, 2.4 Hz, H-6'), 7.57 (1H, d, *J* = 2.4 Hz, H-2'), 7.00 (1H, d, *J* = 8.4 Hz, H-5'), 6.83 (1H, s, H-5), 5.08 (1H, d, *J* = 7.2 Hz, Glc, H-1''), 3.90 (3H, s, 4'-OCH_3), 3.85 (3H, s, 3-OCH_3), 3.77 (3H, s, 7-OCH_3), 3.94 (2H, m, Glc, H-6''), 3.69 (1H, dd, *J* = 12.4, 6.4Hz, Glc, H-3''), 3.56 (1H, m, Glc, H-2''), 3.50 (1H, m, Glc, H-5''), 3.40 (1H, m, Glc, H-4''). ^{13}C-NMR (100MHz, CD_4O) *δ* (ppm): 180.3 (C-4), 158.2 (C-2), 157.9 (C-6), 153.7 (C-8), 153.4 (C-9), 151.8 (C-4'), 147.6 (C-3'), 139.8 (C-3), 133.8 (C-7), 124.0 (C-1'), 122.4 (C-6'), 116.3 (C-2'), 112.3 (C-5'), 108.1 (C-10), 101.9 (Glc, C-1''), 95.5 (C-5), 78.5 (Glc, C-5''), 77.9 (Glc, C-3''), 74.7 (Glc, C-2''), 71.3 (Glc, C-4''), 62.5 (Glc, C-6''), 61.5 (7-OCH_3), 60.5 (3-OCH_3), 56.4 (4'-OCH_3). Compound **9** was identified as 8,3'-dihydroxy-3,7,4'-trimethoxy-6-*O*-*β*-*D*-glucopyranosyl flavone, comparing the physical and spectral data with the reported data [24].

3,5-dihydroxy-3',5'-dimethoxyflavon-7-*O*-*β*-*D*-glucopyranoside (**10**): Yellow amo rphous powder. ^{1}H-NMR (400 MHz, DMSO-d_6) *δ* (ppm): 12.52 (-OH), 9.35 (-OH), 7. 43 (1H, d, *J* = 2.0 Hz, H-2'), 6.97 (1H, d, *J* = 2.0 Hz, H-6'), 6.65 (1H, d, *J* = 2.0 Hz, H-4'), 6.32 (1H, d, *J* = 2.0 Hz, H-8), 5.32 (1H, d, *J* = 2.0 Hz, H-6), 4.51 (1H, d, *J* = 7.0 H z, Glc, H-1''), 3.73 (3'-OCH_3), 3.67 (5'-OCH_3), 3.58 (2H, m, Glc, H-6''), 3.11 (4H, m, H-2''~5''). ^{13}C-NMR (100 MHz, DMSO-d_6) *δ* (ppm): 178.2 (C-4), 162.9 (C-5), 160.9 (C-7), 156.0 (C-5'), 155.9 (C-3'), 150.3 (C-2), 146.3 (C-9), 138.2 (C-3), 122.1 (C-1'), 1 20.5 (C-4'), 115.1 (C-2'), 111.9 (C-6'), 105.9 (C-10), 99.8 (Glc, C-1''), 99.2 (C-6), 94.5 (C-8), 77.1 (Glc, C-3''), 76.3 (Glc, C-5''), 73.1 (Glc, C-2''), 69.5 (Glc, C-4''), 60.6 (Glc, C-6''), 59.8 (3'-OCH_3), 55.6 (5'-OCH_3). Compound **10** was identified as 3,5-dihydroxy -3',5'-dimethoxyflavon-7-*O*-*β*-*D*-glucopyranoside, comparing the physical and spectral data with the reported data [25].

Trifolin (**11**): Yellow amorphous powder. ^{1}H-NMR (400 MHz, CD_4O) *δ* (ppm): 8. 05 (2H, d, *J* = 8.4 Hz, H-2', H-6'), 6.88 (2H, d, *J* = 8.4 Hz, H-3', H-5'), 6.38 (1H, d, *J* = 1.9 Hz, H-8), 6.19 (1H, d, *J* = 1.9 Hz, H-6), 5.24 (1H, d, *J* = 6.8 Hz, Gal, H-1''), 3.82

(1H, d, J = 3.5 Hz, Gal, H-4''), 3.69(1H, dd, J = 11.6, 2.4 Hz, Gal, H-2''), 3.53(1H, dd, J = 12.0, 5.6 Hz, Gal, H-6''a), 3.44 (1H, m, Gal, H-3''), 3.41 (1H, m, Gal, H-6''b), 3.21 (1H, m, Gal, H-5''). ^{13}C-NMR (100 MHz, CD_4O) δ: 179.4 (C-4), 166.4 (C-7), 161.6 (C-5), 159.0 (C-4'), 158.5 (C-2, C-9), 135.5 (C-3), 132.3 (C-2', 6'), 122.8 (C-1'), 116.1 (C-3', C5'), 105.6 (C-10), 104.2 (Gal, C-1), 100.1 (C-6), 94.8 (C-8), 78.4 (Gal, C-5), 78.1 (Gal, C-3), 75.7 (Gal, C-2), 71.4 (Gal, C-4), 62.6 (Gal, C-6). Compound **11** was identified as trifolin, comparing the physical and spectral data with the reported data [26].

3,6-O-dimethylquercetagetin-7-*O*-*β*-*D*-glucoside (**12**): Yellow amorphous powder. ^{1}H-NMR (400 MHz, CD_4O) δ (ppm): 7.62 (1H, d, J = 2.4 Hz, H-2′),7.54 (1H, dd, J = 8.4, 2.0 Hz, H-6′), 6.87 (1H, d, J = 8.4 Hz, H-5′), 6.85 (1H, s, H-8), 5.09 (1H, d, J = 6.8 Hz, Glc, H-1″), 3.92 (1H, dd, J = 12.0, 5.6 Hz, Glc, H-6″a), 3.86 (3H, s, 6-OCH_3), 3.78 (3H, s, 3-OCH_3), 3.70 (1H, dd, J = 12.0, 5.6 Hz, Glc, H-6″b), 3.58 (1H, m Glc, H-3″), 3.53 (1H, m, Glc, H-5″), 3.49 (1H, m Glc, H-2″), 3.40 (1H, m Glc, H-4″); ^{13}C-NMR (100 MHz, CD_4O) δ (ppm): 180.3 (C-4), 158.7 (C-2), 157.8 (C-7), 153.7 (C-5), 153.3 (C-9), 150.1 (C-4′), 146.5 (C-3′), 139.5 (C-3), 133.7 (C-6), 122.7 (C-1′), 122.6 (C-6′), 116.6 (C-2′), 116.4 (C-5′), 108.0 (C-10), 101.9 (Glc, C-1″), 95.5 (C-8), 78.5 (Glc, C-5″), 77.9 (Glc, C-2″), 74.7 (Glc, C-3″), 71.3 (Glc, C-4″), 62.6 (Glc, C-6″), 61.5 (6-OCH_3), 60.5 (3-OCH_3). Compound **12** was identified as 3,6-O-dimethylquercetagetin-7-*O*-*β*-*D*-glucoside, comparing the physical and spectral data with the reported data [27].

3,4'-dimethoxy-7-*O*-*β*-glucopyranoside quercetin (**13**): Yellow amorphous powder. ^{1}H-NMR (400 MHz, DMSO-d_6) δ (ppm): 12.63 (1H, s, 5-OH), 9.96 (1H, s, 3'-OH), 7.63 (1H, d, J = 2.0 Hz, H-2'), 7.59 (1H, dd, J = 8.4, 2.4, Hz, H-6'), 6.94 (1H, d, J = 8.4 Hz, H-5'), 6.82 (1H, d, J = 2.4 Hz, H-8), 6.42 (1H, d, J = 2.0 Hz, H-6), 5.03 (1H, d, J = 7.2 Hz, Glc, H-1''), 3.83 (3H, s, 4′-OCH_3), 3.79 (3H, s, 3-OCH_3), 3.68 (1H, dd, J = 10.4, 5.2 Hz, Glc, H-6″a), 3.45 (1H, m Glc, H-3″), 3.42 (3H, m, Glc, H-2'', 4'', 6″b), 3.27 (1H, m, Glc, H-5″); ^{13}C-NMR (100 MHz, DMSO-d_6) δ (ppm): 178.2 (C-4), 163.0 (C-7), 160.9 (C-5), 156.1 (C-9), 156.0 (C-2), 150.0 (C-4'), 147.5 (C-3'), 138.0 (C-3), 122.4 (C-1'), 120.7 (C-6'), 115.7 (C-2'), 112.1 (C-5'), 105.9 (C-10), 100.0 (Glc, C-1″), 99.2 (C-6), 94.9 (C-8), 77.3 (Glc, C-3″), 76.5 (Glc, C-5″), 73.2 (Glc, C-2″), 69.6 (Glc, C-4″), 60.6 (Glc, C-6″), 59.8 (3-OCH_3), 55.8 (4'-OCH_3). Compound **13** was identified as 3,4'-dimethoxy-7-*O*-*β*-glucopyranoside quercetin, comparing the physical and spectral data with the reported data [28].

5,3'-dihydroxyl-3,6,4'-trimethoxylflavone-7-*O*-*β*-*D*-glucopyranoside (**14**): Yellow amorphous powder. ^{1}H-NMR (400 MHz, CD_4O) *δ* (ppm): 7.67 (1H, dd, *J* = 8.4, 2.0 H z, H-6'), 7.62 (1H, d, *J* = 2.0 Hz, H-2'), 7.06 (1H, d, *J* = 8.8 Hz, H-5'), 6.91 (1H, s, H-8), 5.12 (1H, d, *J* = 7.2 Hz, Glc, H-1''), 3.94 (3H, s, 4'-OCH_3), 3.89 (3H, s, 3-OCH_3), 3. 82 (3H, s, 6-OCH_3), 3.72 (1H, dd, *J* = 12.0, 6.0 Hz, Glc, H-5''), 3.62 (2H, m, Glc, H-6' '), 3.55 (1H, t, *J* = 8.5 Hz, Glc, H-3''), 3.42 (2H, m, Glc, H-2'', 4''). ^{13}C-NMR (100 MH z, CD_4O) *δ* (ppm): 180.3 (C-4), 158.3 (C-2), 157.8 (C-7), 153.7 (C-5), 153.3 (C-9), 15 1.9 (C-4'), 147.7 (C-3'), 139.8 (C-3), 133.8 (C-6), 124.0 (C-1'), 122.3 (C-6'), 116.3 (C-2'), 112.2 (C-5'), 108.1 (C-10), 101.9 (Glc, C-1''), 95.5 (C-8), 77.9 (Glc, C-5''), 78.4 (G lc, C-3''), 74.7 (Glc, C-2''), 71.2 (Glc, C-4''), 62.5 (Glc, C-6''), 61.4 (6-OCH_3), 60.5 (3-OCH_3), 56.3 (4'-OCH_3). Compound **14** was identified as 5,3'-dihydroxyl-3,6,4'-trimeth oxylflavone-7-*O*-*β*-*D*-glucopyranoside, comparing the physical and spectral data with the reported data [29].

Luteolin (**15**): Yellow amorphous powder. ^{1}H-NMR (400 MHz, DMSO-d_6) *δ* (pp m): 13.18 (1H, s, 5-OH), 7.57 (1H, dd, *J* = 8.2, 2.2 Hz, H-6'), 7.54 (1H, d, *J* = 2.2 Hz, H-2'), 7.01(1H, d, *J* = 8.2 Hz, H-5'), 6.80 (1H, s, H-3), 6.57 (1H, d, *J* = 1.9 Hz, H-8), 6. 30 (1H, d, *J* = 1.9 Hz, H-6); ^{13}C-NMR (100 MHz, DMSO-d_6) *δ* (ppm): 181.5 (C-4), 16 5.2 (C-2), 163.9 (C-7), 161.5 (C-5), 157.5 (C-9), 150.5 (C-4'), 146.1 (C-3'), 121.2 (C-1 '), 119.1 (C-6'), 116.1 (C-5'), 113.1(C-2'), 103.4 (C-10), 102.6 (C-3), 99.2 (C-6), 94.0 (C-8). Compound **15** was identified as Luteolin, comparing the physical and spectral d ata with the reported data [30].

2-(S)-isookanin-4'-methoxy-8-*O*-*β*-*D*-glucopyranoside (**16**): Yellow amorphous p owder. ^{1}H-NMR (400 MHz, CD_4O) *δ* (ppm): 7.59 (1H, d, *J* = 8.8 Hz, H-5), 7.13 (1H, d, *J* = 2 Hz, H-2′), 6.99 (1H, dd, *J* = 8.4, 2.0 Hz, H-6′), 6.94 (1H, d, *J* = 8.0 Hz, H-5′), 6.59 (1H, d, *J* = 8.8 Hz, H-6), 5.49 (1H, dd, *J* = 12.0, 3.0 Hz, H-2), 4.80 (1H, d, *J* = 7.6 Hz, Glc, H-1″), 3.87 (3H, s, 4′-OCH_3), 3.83 (1H, dd, *J* = 12.0, 2.4 Hz, Glc, H-6″a), 3. 74 (1H, dd, *J* = 12.0, 4.0 Hz, Glc, H-6″b), 3.50 (1H, m, Glc, H-2″), 3.43 (1H, m, Glc, H-3″), 3.38 (1H, m, Glc, H-4″), 3.26 (1H, m, Glc, H-5″), 3.02 (1H, dd, *J* = 12.0, 3.2 H z, H-3b), 2.82 (1H, m, H-3a); ^{13}C-NMR (100 MHz, CD_4O) *δ* (ppm): 193.1 (C-4), 158. 9 (C-7), 157.0 (C-9), 149.1 (C-4′), 147.7 (C-3′), 134.1 (C-8), 133.4 (C-1′), 124.9 (C-5), 118.7 (C-6′), 116 (C-10),114.3 (C-2′), 112.7 (C-5′), 111.7 (C-6), 106.8 (Glc, C-1″), 80. 9 (C-2), 78.4 (Glc, C-5″), 77.6 (Glc, C-3″), 75.4 (Glc, C-2″), 70.7 (Glc, C-4″), 62.0 (G

lc, C-6″), 56.4 (4′-OCH_3), 45.1 (C-3). Compound **16** was identified as 2-(S)-isookanin -4'-methoxy-8-*O*-*β*-*D*-glucopyranoside, comparing the physical and spectral data with the reported data [31].

Hesperedin-7-*O*-*β*-*D*-glucopyranoside (**17**): Yellow amorphous powder. ^{1}H-NMR (400 MHz, DMSO-d_6) δ (ppm): 12.01 (1H, s, 5-OH), 9.15 (1H, s, 3'-OH), 7.05 (1H, d, J = 2.0 Hz, H-5'), 6.87 (1H, d, J = 1.6 Hz, H-2'), 6.73 (1H, d, J = 8.4 Hz, H-6'), 6.10 (1 H, d, J = 2.4 Hz, H-6), 6.08 (1H, s, H-8), 5.43 (1H, dd, J = 12.8, 2.8 Hz, H-2), 5.02 (1 H, d, J = 5.2 Hz, Glc, H-1"), 3.73 (3H, s, 4'-OCH_3), 3.60 (2H, m, Glc, H-6"), 3.41 (1H, dd, J = 17.4, 12.0 Hz, H-3b), 3.40 (1H, m, Glc, H-5"), 3.23 (1H, m, Glc, H-3"), 3.13 (2H, m, Glc, H-2", 4"), 2.71 (1H, dd, J = 10.4, 3.2 Hz, H-3a). ^{13}C-NMR (100 MHz, D MSO-d_6) δ (ppm): 197.4 (C-4), 165.4 (C-7), 162.9 (C-5), 162.8 (C-9), 147.6 (C-4'), 14 7.1 (C-3'), 129.2 (C-1'), 115.2 (C-6'), 112.0 (C-2'), 111.3 (C-5'), 103.3 (C-10), 99.7 (Gl c, C-1"), 96.6 (C-6), 95.5 (C-8), 78.5 (C-2), 77.1 (Glc, C-3"), 76.3 (Glc, C-5"), 73.1 (G lc, C-2"), 69.5 (Glc, C-4"), 60.6 (Glc, C-6"), 55.7 (4'-OCH_3), 42.2 (C-3). Compound **1 7** was identified as hesperedin-7-*O*-*β*-*D*-glucopyranoside, comparing the physical and spectral data with the reported data [32].

Maritimein (**18**): Orange red amorphous powder. ^{1}H-NMR (400 MHz, DMSO-d_6) δ (ppm): 7.47 (1H, d, J = 1.2 Hz, H-2'), 7.34 (1H, dd, J = 8.4, 2.0 Hz, H-6'), 7.22 (1H, d, J = 8.4 Hz, H-4), 7.07 (1H, d, J = 8.8 Hz, H-5), 6.87 (1H, d, J = 8.0 Hz, H-5'), 6.72 (1H, s, H-10), 4.95 (1H, d, J = 7.6 Hz, Glc, H-1"), 3.52 (2H, m, Glc, H-6"), 3.36～3.4 6 (3H, overlapped, Glc, H-2", 3", 4"), 3.17 (1H, m, Glc, H-5"); ^{13}C-NMR (100 MHz, D MSO-d_6) δ (ppm): 182.5 (C-3), 154.1 (C-8), 152.3 (C-6), 148.4 (C-4'), 145.6 (C-2), 14 5.5 (C-3'), 132.4 (C-7), 125.0 (C-6'), 123.4 (C-1'), 118.4 (C-2'), 117.2 (C-9), 116.1 (C- 5'), 114.6 (C-4), 113.0 (C-10), 112.0 (C-5), 101.6 (Glc, C-1"), 77.4 (Glc, C-3"), 75.8 (G lc, C-5"), 73.3 (Glc, C-2"), 69.7 (Glc, C-4"), 60.7 (Glc, C-6"). Compound **18** was ident ified as maritimein, comparing the physical and spectral data with the reported data [33].

(Z)-6-*O*-(6″-acetyl-*β*-*D*-glucopyranosyl)-7,3′,4′-tetrahydroxy-aurone (**19**): Orang e red amorphous powder. ^{1}H-NMR (400 MHz, DMSO-d_6) δ (ppm): 7.56 (1H, d, J = 1. 8 Hz, H-2'), 7.38 (lH, dd, J = 8.4 Hz, H-6'), 7.24 (lH, d, J = 8.4 Hz, H-4), 7.05 (lH, d, J = 8.4 Hz, H-5), 6.85 (lH, d, J = 8.8 Hz， H-5'), 6.78 (1H, s, H-10), 5.12 (1H, d, J = 7.

6, H-1''), 4.46 (1H, dd, J = 12.0, 2.4 Hz, Glc, H-6''a), 4.28 (1H, dd, J = 12.0, 6.8 Hz, Glc, H-6''b), 3.41~3.59 (4H, overlapped, Glc, H-2'', 3'', 4'', 5''), 2.09 (3H, s, -OAc); ^{13}C-NMR (100 MHz, DMSO-d_6) δ (ppm): 185.6 (C-3), 172.7 (-OAc), 156.4 (C-8), 153.8 (C-6), 149.8 (C-4'), 147.5 (C-2), 146.8 (C-3'), 135.1 (C-7), 126.9 (C-6'), 125.4 (C-1'), 119.3 (C-2'), 119.1 (C-9), 116.7 (C-5'), 115.6 (C-10), 115.2 (C-4), 113.4 (C-5), 102.9 (Glc, C-1''), 77.3 (Glc, C-3''), 75.6 (Glc, C-5''), 74.7 (Glc, C-2''), 71.5 (Glc, C-4''), 64.6 (Glc, C-6''), 20.8 (-OAc). Compound **19** was identified as (Z)-6-*O*-(6″-acetyl-*β*-*D*-glucopyranosyl)-7,3′,4′-tetrahydroxy-aurone, comparing the physical and spectral data with the reported data [34].

7-phenyl-hepta-4,6-diyn-1,2-diol (**21**): Yellow amorphous powder. ^{1}H-NMR (400 MHz, CD_4O) δ (ppm): 7.48~7.52 (2H, m, H-2', H-6'), 7.27~7.37 (3H, m, H3', H-4', H-5'), 3.97 (1H, m, H-2), 3.78 (1H, dd, J = 11.1, 3.6 Hz, H-1a), 3.66 (1H, dd, J = 11.1, 6.6 Hz, H-1b), 2.65 (2H, d, J = 6.6 Hz, H2-3); ^{13}C-NMR (100 MHz, CD_4O) δ (ppm): 133.7 (C2'), 133.5 (C-6'), 130.2 (C-3'), 129.7 (C-5'), 127.6 (C-4'), 123.2 (C-1'), 82.2 (C-4), 75.6 (C-7), 75.1 (C-5), 71.6 (C-2), 67.2 (C-6), 66.0 (C-1), 25.2 (C-3). Compound **21** was identified as 7-phenyl-hepta-4,6-diyn-1,2-diol, comparing the physical and spectral data with the reported data [3].

7-phenyl-hepta-4,6-diyne-2-ol (**22**): Yellow oil. ^{1}H-NMR (400 MHz, $CDCl_3$) δ (ppm): 7.52 (dd, 2H, J = 7.7, 1.2 Hz, H-2', H-6'), 7.37 (m, 1H, J = 7.2, 1.2 Hz, H-4'), 7.32 (dd, 2H, J = 7.7, 7.2 Hz, H-3', H-5'), 4.05 (s, 3H, H-2), 2.66 (s, 3H, H-3), 1.38 (s, H, H-1); ^{13}C-NMR (100 MHz, $CDCl_3$) δ (ppm): 132.5 (C-2', C-6'), 129.1 (C-4'), 128.4 (C-3', C-5'), 121.7 (C-1'), 80.7 (C-4), 75.4 (C-7), 73.9 (C-5), 67.4 (C-6), 66.4 (C-2), 30.1 (C-3), 22.5 (C-1). Compound **22** was identified as 7-phenyl-hepta-4,6-diyne-2-ol, comparing the physical and spectral data with the reported data [35].

1-phenylhepta-1,3,5-triyne (**23**): Yellowish-brown acicular crystal. ^{1}H-NMR (400 MHz, $CDCl_3$) δ (ppm): 7.52 (dd, 2H, J = 7.7, 1.2 Hz, H-2', H-6'), 7.36 (dd, 1H, J = 7.1, 1.3 Hz, H-4'), 7.32 (dd, 2H, J = 7.8, 7.1 Hz, H-3', H-5'), 2.03 (m, 3H, H-7); ^{13}C-NMR (100 MHz, $CDCl_3$) δ (ppm): 133.0 (C-2', C-6'), 129.5 (C-4'), 128.5 (C-3', C-5'), 121.1 (C-1'), 78.3 (C-6), 75.2 (C-1), 74.6 (C-2), 67.4 (C-5), 64.9 (C-4), 58.9 (C-3), 4.7 (C-7). Compound **23** was identified as 1-phenylhepta-1,3,5-triyne, comparing the physical and spectral data with the reported data [36].

icthyothereol acetate (**24**): Brown oil. ^{1}H-NMR (400 MHz, CD_4O) δ (ppm): 6.22 (dd, 1H, J = 16.0, 5.6 Hz, H-1'), 5.81 (dd, 1H, J = 16.3 Hz，H-2'), 4.61 (m, 1H, H-2), 3.96 (m, 1H, H-5), 3.74 (ddd, 1H, J = 9.6, 5.4, 1.8 Hz, H-1), 2.21 (m, 1H, H-3), 2.08 (s, 3H, H-3''), 2.01 (s, 3H, H-9'), 1.76 (m, 2H, H-4), 1.52 (dd, 1H, J = 11.0, 5.2 Hz, H-3'); ^{13}C-NMR (100 MHz, CD_4O) δ (ppm): 171.8 (C-2''), 146.3 (C-1'), 110.9 (C-2'), 80. 2 (C-1), 79.7 (C-8'), 75.8 (C-4'), 74. 4 (C-3'), 72.8 (C-2), 68.5 (C-5), 68.4 (C-6'), 65.0 (C-7'), 59.2 (C-5'), 30.2 (C-3), 26.0 (C-4), 21.0 (C-3''), 3.9 (C-9'). Compound **24** was i dentified as icthyothereol acetate, comparing the physical and spectral data with the re ported data [37].

3-*β*-*D*-Glucopyranosyloxy-1-hydroxy-6(E)-tetradecene-8,10,12-triyne (**25**): Yello w crystals. ^{1}H-NMR (400 MHz, CD_4O) δ (ppm): 6.44 (1H, dt, J = 15.6, 6.8 HZ, H-6), 5.64 (1H, d, J = 16.0 HZ, H-7), 4.60 (1H, d, J = 7.8 HZ, H-1'), 4.20 (1H, dd, J = 11.8, 2.7 Hz, H-6a'), 3.71 (1H, m, H-3), 3.49-3.56 (2H, m, H-1), 3.07-3.15 (2H, m, H-5), 2.2 2-2.42 (2H, m, H-4), 2.15-3.33 (5H, overlapped, H-2'~H-5', H-6b'), 1.91 (3H, s, H-14), 1.53 (2H, m, H-2); ^{13}C-NMR (100 MHz, CD_4O) δ (ppm): 151.8 (C-6), 109.2 (C-7), 1 03.9 (C-1'), 79.0 (C-13), 78.12 (C-3), 77.8 (C-3'), 76.8 (C-5'), 75.5 (C-12), 75.3 (C-2'), 73.6 (C-1), 72.2 (C-4'), 67.0 (C-10), 65.1 (C-9), 63.2 (C-6'), 59.6 (C-8), 59.1 (C-1), 3 9.1 (C-2), 35.1 (C-4), 30.0 (C-5), 3.9 (C-14). Compound **25** was identified as 3-*β*-*D*-G lucopyranosyloxy-1-hydroxy-6(E)-tetradecene-8,10,12-triyne, comparing the physical and spectral data with the reported data [38].

2-*β*-*D*-glucopyranosytoxy-1-hydroxy-5(E)-tridecene-7,9,11-triyne (**26**): Yellow cr ystals. ^{1}H-NMR (400 MHz, CD_4O) δ (ppm): 6.39 (1H, dt, J = 16.0, 7.2 HZ, H-5), 5.61 (1H, d, J = 16.0 HZ, H-6), 4.28 (1H, d, J = 8 HZ, H-1'), 3.82 (1H, dd, J = 11.6, 1.6 H z, H-6a'), 3.1-3.64 (5H, m, H-2'~H-5', H-6b'), 3.65 (1H, m, H-2), 2.21-2.40 (2H, m, H-4), 1.53-1.7 (2H, m, H-3), 1.94 (3H, s, H-13), 1.67 (2H, m, H-1); ^{13}C-NMR (100 MHz, CD_4O) δ (ppm): 151.5 (C-6), 109.4 (C-7), 104.4 (C-1'), 82.2 (C-3), 79.1 (C-13), 77.9 (C-3'), 77.8 (C-5'), 75.5 (C-11), 75.1 (C-2'), 73.6 (C-12), 71.5 (C-4'), 67.1 (C-10), 65.8 (C-1), 65.2 (C-9), 62.6 (C-6'), 59.6 (C-8), 31.4 (C-4), 30.0 (C-5), 3.9 (C-14). Compou nd **26** was identified as 2-*β*-*D*-glucopyranosytoxy-1-hydroxy-5(E)-tridecene-7,9,11-tri yne, comparing the physical and spectral data with the reported data [39].

2-*β-D*-glucopyranosyloxy-1-hydroxytrideca-5,7,9,11-tetrayne (**27**): Yellow crystals. ^{1}H-NMR (400 MHz, CD_4O) δ (ppm): 4.33 (1H, d, *J* = 7.6 HZ, H-1'), 3.85 (1H, dd, *J* = 12.0, 2.4 Hz, H-6a'), 3.74 (1H, dd, *J* = 12.0, 5.6 Hz, H-6b'), 3.59 (2H, m, H-1), 3.62 (1H, m, H-2), 3.25 (4H, m, H-2'~H-5'), 2.58 (2H, m, H-4), 1.99 (3H, s, H-13), 1.77 (2H, m, H-3); ^{13}C-NMR (100 MHz, CD_4O) δ (ppm): 104.8 (C-1'), 81.6 (C-2), 81.56 (C-11), 78.0 (C-12), 77.9 (C-3'), 77.9 (C-5'), 75.2 (C-2'), 71.5 (C-4'), 66.2 (C-5), 65.7 (C-1), 65.0 (C-10), 62.6 (C-6'), 62.4 (C-9), 61.7 (C-6), 60.9 (C-8), 60.1 (C-7), 31.4 (C-3), 16.1 (C-4), 3.83 (C-13). Compound **27** was identified as 2-*β-D*-glucopyranosyloxy-1-hydroxytrideca-5,7,9,11-tetrayne, comparing the physical and spectral data with the reported data [40].

(3S,4S)-(5E)-trideca-1,5-dien-7,9,11-triyne-3,4-diol-4-*O-β-D*-glucopyranoside (**28**): Yellowish oil. ^{1}H-NMR (400 MHz, CD_4O) δ (ppm): 6.41 (1H, dd, *J* = 17.2, 1.8 HZ, H-5), 5.92 (1H, d, *J* = 17.2 HZ, H-6), 5.93 (1H, m, H-2), 5.34 (1H, dd, *J* = 17.2, 4.8 HZ, H-1a), 5.23 (1H, dd, *J* = 10.8, 4.8 HZ, H-1b), 4.59 (1H, d, *J* = 7.6 HZ, H-1'), 4.30 (1H, m, H-3), 4.32 (1H, m, H-4), 3.85 (1H, m, H-6a'), 3.27-3.69 (5H, m, H-2'~H-5', H-6b'), 1.99 (3H, s, H-13); ^{13}C-NMR (100 MHz, CD_4O) δ (ppm): 146.7 (C-5), 137.5 (C-2), 117.2 (C-1), 111.3 (C-6), 103.9 (C-1'), 83.6 (C-4), 79.5 (C-12), 78.0 (C-3'), 77.9 (C-5'), 75.7 (C-10), 75.3 (C-2'), 74.9 (C-3), 74.9 (C-11), 71.4 (C-4'), 67.8 (C-9), 65.1 (C-8), 62.6 (C-6'), 59.4 (C-7), 3.90 (C-13). Compound **28** was identified as (3S,4S)-(5E)-trideca-1,5-dien-7,9,11-triyne-3,4-diol-4-*O-β-D*-glucopyranoside, comparing the physical and spectral data with the reported data [41].

(+)-(8S,9R,10S)-10-methoxyheptadeca-1-ene-11,13-diyne-8,9-diol (**29**): Yellow oil. ^{1}H-NMR (400 MHz, $CDCl_3$) δ (ppm): 5.80-5.88 (1H, m, H-2), 5.17 (1H, dd, *J* = 17.2, 4.8 HZ, H-1b), 4.99 (1H, dd, *J* = 10.8, 4.8 HZ, H-1a), 4.54 (1H, d, *J* = 7.2 HZ H-10), 3.69 (1H, m, H-8), 3.56 (1H, m, H-9), 3.47, (3H, s, $-OCH_3$), 2.12 (2H, t, *J* = 7.2 Hz H-15), 1.90 (2H, m, *J* = 6.9 Hz, H-3), 1.59 (2H, m, H-16), 1.2 (8H, m, H-4~H-7), 0.89 (3H, s, H-17); ^{13}C-NMR (100 MHz, $CDCl_3$) δ (ppm): 137.4 (C-2), 116.1 (C-1), 80.2 (C-14), 75.7 (C-10), 73.5 (C-12), 73.5 (C-9), 70.6 (C-8), 69.7 (C-11), 61.6 (C-13), 56.2 ($-OCH_3$), 31.8 (C-3), 30.8 (C-7), 29.8 (C-5), 29.7 (C-4), 25.7 (C-6), 22.8 (C-16), 21.0 (C-15), 14.2 (C-17). Compound **29** was identified as (+)-(8S,9R,10*S*)-10-methoxyheptadeca-1-ene-11,13-diyne-8,9-diol, comparing the physical and spectral data with the reported data [42].
